# Hidden Markov Model Analysis of Maternal Behavior Patterns in Inbred and Reciprocal Hybrid Mice

**DOI:** 10.1371/journal.pone.0014753

**Published:** 2011-03-08

**Authors:** Valeria Carola, Olivier Mirabeau, Cornelius T. Gross

**Affiliations:** 1 Mouse Biology Unit, European Molecular Biology Laboratory (EMBL), Monterotondo, Italy; 2 Santa Lucia Foundation, European Centre for Brain Research (CERC), Rome, Italy; University of Queensland, Australia

## Abstract

Individual variation in maternal care in mammals shows a significant heritable component, with the maternal behavior of daughters resembling that of their mothers. In laboratory mice, genetically distinct inbred strains show stable differences in maternal care during the first postnatal week. Moreover, cross fostering and reciprocal breeding studies demonstrate that differences in maternal care between inbred strains persist in the absence of genetic differences, demonstrating a non-genetic or epigenetic contribution to maternal behavior. In this study we applied a mathematical tool, called hidden Markov model (HMM), to analyze the behavior of female mice in the presence of their young. The frequency of several maternal behaviors in mice has been previously described, including nursing/grooming pups and tending to the nest. However, the ordering, clustering, and transitions between these behaviors have not been systematically described and thus a global description of maternal behavior is lacking. Here we used HMM to describe maternal behavior patterns in two genetically distinct mouse strains, C57BL/6 and BALB/c, and their genetically identical reciprocal hybrid female offspring. HMM analysis is a powerful tool to identify patterns of events that cluster in time and to determine transitions between these clusters, or hidden states. For the HMM analysis we defined seven states: arched-backed nursing, blanket nursing, licking/grooming pups, grooming, activity, eating, and sleeping. By quantifying the frequency, duration, composition, and transition probabilities of these states we were able to describe the pattern of maternal behavior in mouse and identify aspects of these patterns that are under genetic and nongenetic inheritance. Differences in these patterns observed in the experimental groups (inbred and hybrid females) were detected only after the application of HMM analysis whereas classical statistical methods and analyses were not able to highlight them.

## Introduction

Natural variation in the amount and type of maternal care received is associated with differences in adult behavioral traits in a wide range of mammalian species. Although much of this association depends on the fact that mothers and their offspring share significant genetic variation, cross fostering and reciprocal breeding experiments in rodents have shown that non-genetic or epigenetic mechanisms also play an important part in this association [1], [2]. In mice, the study of maternal effects on the offspring has been aided by the existence of inbred strains that show large and stable differences in maternal care. Mothers of the C57BL/6 inbred strain, for example, exhibit more licking and grooming of pups and arched-back nursing compared to the BALB/c strain [3]–[11] and offspring of these two strains show differences in adult behavior that can at least partially be traced back to their differences in maternal care [10], [12]–[14]. Maternal non-genetic or epigenetic mechanisms also contributes to maternal behavior itself, with female offspring of cross fostering or reciprocal breeding between C57BL/6 and BALB/c parents showing maternal behavior that reflects the behavior of their mother [3], [4], [6], [10], [15], [16].

The frequency of several maternal behaviors in laboratory mice has been previously described, including nursing and grooming pups, tending to the nest, and feeding [7], [9], [10], [11]. However, the ordering, clustering, and transitions between these behaviors have not been systematically described and thus a global description of maternal behavior is lacking. A powerful tool for the analysis of sequential patterns of events is the hidden Markov model (HMM) formalism [17]. Although used extensively for the analysis of speech and hand gestures, as well as DNA and protein sequences [18]–[21] HMM has been rarely used to analyse behavioral mouse data. It has been applied to the analysis of locomotor patterns in several behavioral tests [22], [23]. HMM can provide information both about which observed behavioral variables are sequentially associated as well as the frequencies of transitions between these associated behaviors, or “states”.

Here, we used HMM to define and characterize states that describe the maternal behavior patterns of C57BL/6 and BALB/c mice and their reciprocal hybrid offspring. Our analysis identified gross differences in behavioral repertoires and trajectories that included differences in the frequency, composition, and transitions between identified behavioral states. Surprisingly, only a limited subset of these differences in maternal behavior were observed in reciprocal hybrid offspring, suggesting that the maternal effect on this behavior is limited to critical elements of the maternal strategy. However these differences were detected after HMM analysis and were not really observable with classical statistical methods. We therefore demonstrate the power of HMM to extract behavioral patterns that are not revealed by simple frequency analysis alone and provide a detailed description of the maternal effect on behavior in mice.

## Materials and Methods

### Animals

Three week old C57BL/6J@Ico (C57BL/6) and BALB/cByJ@Ico (BALB/c) male and female mice were purchased from Charles River Laboratories (Calco, Italy). Reciprocal F1 hybrid females were obtained by breeding C57BL/6 females and BALB/c males (B6xC mothers) and BALB/c females and C57BL/6 males (CxB6 mothers). To reduce differences in maternal behavior due to genetic differences of their offspring, maternal behavior of inbred mothers was assessed in C57BL/6 (N = 26; litter size = 6.33±0.55) and BALB/c (N = 26; litter size = 5.99±0.59) females mated at 8 weeks of age with BALB/c and C57BL/6 males, respectively. Maternal behavior of reciprocal F1 hybrid mothers was assessed in B6xC (N = 36; litter size = 6.85±0.47) and CxB6 (N = 39; litter size = 6.74±0.52) females mated at 10–12 weeks of age with C57BL/6 males.

For all breeding, fathers were removed before parturition and mothers and offspring left undisturbed and without cage changing until postnatal day 21 at which time pups were weaned, housed 3–5 per cage, and weekly cage cleaning was resumed. No special effort was made to normalize litter sex composition or size. Food and water were provided *ad libitum*, and mice were housed on a 12 h light/dark cycle with lights on at 7:00 AM. All animals were handled in strict accordance with good animal practice as defined by the relevant national and local animal welfare bodies. All experiments of this study were approved by the ethics committee of the Italian Department of Health and therefore conducted under license/approval ID #:91/2007-B, according with Italian regulations on the use of animals for research (legislation DL 116/92) and NIH guidelines on animal care.

### Behavioral observation

Maternal observations (60 observations/hour) were performed for 3 h during the light period (10:00–11:00 AM, 2:00–3:00 PM, and 6:00–7:00 PM) and 1 h during the night period (7:00–8:00 PM) each day from postnatal day 1 to 19. All observations were conducted by V.C. The following behaviors were scored in the nest: *arched-back nursing* (mother is immobile and in a upright arching posture with rigid limbs placed directly underneath or slightly away from the body, head depressed, and with all or most pups attached to the nipples), *arched-back nursing*<*half litter* (more than half of pups outside nest), *blanket nursing* (mother is less immobile and in a low arching posture with some rigid limbs or lying flat on the pups with little or no limb support), *blanket nursing<half litter* (more than half of pups outside nest), *licking/grooming pups*, *licking/grooming pups*<*half litter*, *nest building*, *self grooming*, *sniffing pups*, and *sniffing nest*. *Time in nest* was calculated by summing time spent in all these states. The following behaviors were scored outside the nest: *carrying pups* (retrieval of pups form outside nest), *carrying tail*, *climbing* (on the cage top), *digging*, *drinking*, *eating*, *moving pups*, *rearing*, *self-grooming*, *sniffing cage*, and *sleeping*.

### Statistics

The effect of the hour of the day and strain on behavioral measures was first analyzed by multiple analysis of variance (MANOVA) followed by univariate ANOVA and in cases of significance (P<0.05), post-hoc comparisons using Duncan's test. The effects of postnatal day and interaction between strain and postnatal day were analyzed by repeated measure ANOVA. Analysis of covariance (ANCOVA) was performed to evaluate if the litter size and sex ratio affect/influence the strain effect observed/detected. All statistical analyses were carried out with the help of Statistica (StatSoft, Tulsa, OK) and SPSS (SPSS, Chicago, IL) software.

### HMM analysis and statistics

Sequences of observed behaviors for the training of HMM states were defined as consecutive behavioral observations taken once a minute for one hour. A hidden Markov model (HMM) is characterized by its state transition probability matrix *A* (chance to go from one state to another), its observation matrix *B* (contribution of each behavior to each state), and initial state probability matrix *π* (chance of being in a given state at the start of a sequence). In our analysis we defined seven HMM states: blanket nursing (BLN), arched-back nursing (ABN), licking/grooming pups (LG), grooming (GRO), eating (EAT), activity (ACT) and sleeping (SLP). A single HMM was derived by applying the Baum-Welch algorithm to observed behavioral sequences from mothers of all four strains starting from an initial HMM (*A*_0_, *B*_0_, *π*_0_). This algorithm is an iterative procedure in which the HMM matrices are re-estimated at each step *n* to increase the fit between the model and the data until it reaches an optimum [17]. The final HMM, MatHMM (*A*_end_, *B*_end_, *π*_end_) that corresponded to the optimal fit was used for our analysis. We tested the robustness of this MatHMM to the choice of the initial model. First we tested whether the final model matHMM depended on our choice of initial parameters (*A*_0_, *B*_0_, *π*_0_). For this we ran the Baum-Welch algorithm on the data from all four strains for 100 different initial models where all state transition, observation and initial state probabilities were assigned a random number between 0 and 1 following a uniform distribution. Next, to test whether the choice of seven states lead to the best model we computed the Bayesian Information criterion (BIC) [24] for the model MatHMM and models in which the two states ABN and BLN were merged and/or the two states GRO and LG were merged. Briefly the BIC states that the best model is the one which minimizes the following quantity:
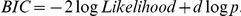
where *p* is proportional to the size of the data, *d* is the number of free parameters in the family of models and *Likelihood* is the likelihood of the data given the HMM, as computed through the forward-backward procedure [17]. In the case of Hidden Markov models, *d* can be calculated as follows:
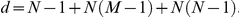
where *N* is the number of states and *M* is the number of distinct behavioral variables.

Finally, MatHMM was used in conjunction with the Viterbi algorithm to assign HMM states to each observed behavioral observation. The labeling data was then used to calculate the frequencies, compositions, and transition probabilities among HMM states for each of the four strains. Significant effects of strain were assessed using standard t tests (state frequencies) or binomial tests (state compositions and transitions). The binomial test was conducted as follows: Let *f_1_* and *f_2_* be frequencies related to states 1 and 2, respectively, and *N_1_* and *N_2_* the number of occurrences. Let 
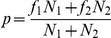
.

If *f_1_N_1_, f_2_N_2_,(N_1_−x_1_)* and *(N_2_−x_2_)* are all larger than 5, then the statistic 
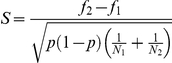
 follows a Normal law U(0, 1). Correction for multiple observations was conducted using the Benjamini Hochberg procedure [25]. Briefly this procedure aims to control the false discovery rate (proportion of erroneous calls among all calls) in multiple statistical testing. Let 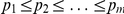 be the ordered observed p-values for each series of tests (e.g. tests of difference in transition probabilities between any two states) and *q* a false discovery rate threshold. If the set 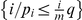 is not empty then define 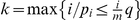. In this case the *k* most significant tests are called significant at the level *q*. Throughout the paper, *q* was set to 0.05.

## Results

### Maternal behavior in inbred and F1 hybrid mice

In order to identify the period of the day (hour) where the highest amount of maternal care/behavior was observable in all four mouse strains, preliminary MANOVA of *arched-back nursing*, *blanket nursing*, and *licking/grooming pups* behaviors was performed. This analysis showed a main effect of hour (λ = 0.831, F [3, 9] = 29.26, P = 0.001) for all behaviors analysed. Univariate results showed (**Figure S1**) that the first two hours of recording were different from the last two hours, with the highest amount of maternal behavior observable during the first phase of the daily observation. The following analyses were therefore performed on the behaviors recorded during the first two hours of the day.

**Figure 1:**
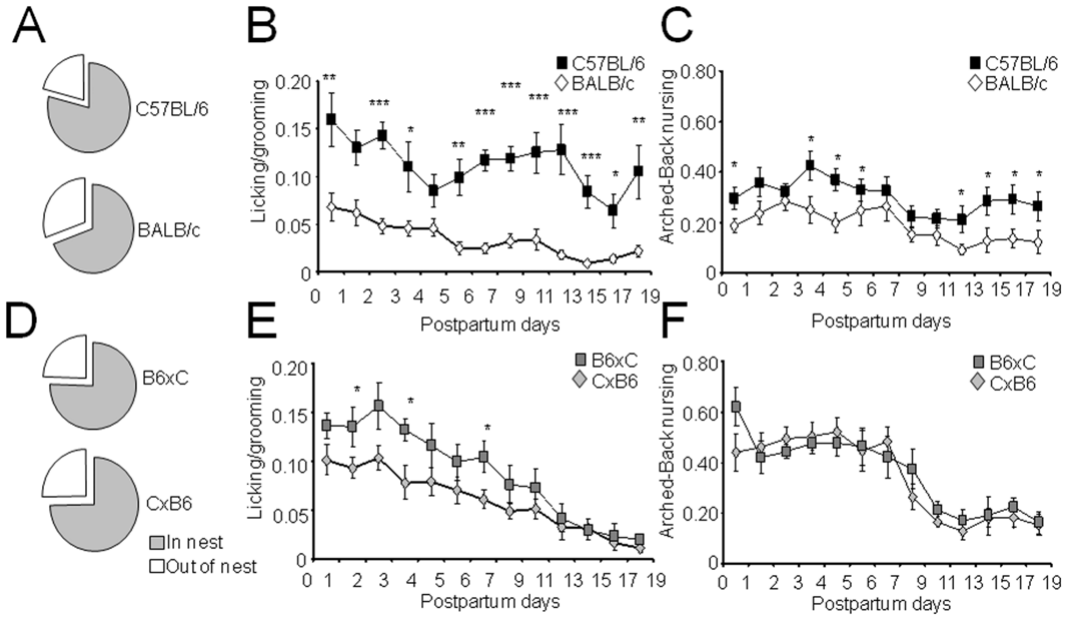
Observed maternal behavior in inbred and reciprocal hybrid mothers. Daily observations of maternal behavior from birth to weaning revealed significantly increased frequency of (**A**) time in nest, (**B**) licking/grooming pups, and (**C**) arched-back nursing by C57BL/6 vs. BALB/c mothers. For reciprocal hybrid mothers, significantly increased (**E**) licking/grooming pups, but not (**D**) time in nest, or (**F**) arched-back nursing was seen in B6xC vs. CxB6 mothers (C57BL/6, N = 26; BALB/c, N = 26; B6xC, N = 36; CxB6, N = 39; * P<0.05, ** P<0.01, *** P<0.001).

Two ANOVAs were performed on the *number of pups* of each litter. No effect of the strain was observed for this variable quantified in both inbred and F1 hybrid mothers. ANOVA of *time in nest* revealed a main effect of strain for inbred mothers (F [1, 50] = 34.3, P = 0.001) with C57BL/6 mothers showing more *time in nest* than BALB/c mothers (**Figure 1A**). No effect of strain was observed for the same variables measured in F1 hybrid mothers (**Figure 1D**). For inbred mothers, MANOVA of behavioral variables (see **Table 1**) revealed a significant main effect of strain (λ = 0.103, F [20, 31] = 13.5, P = 0.001). Univariate results (see **Table 1**) showed that C57BL/6 mothers performed more *arched-back nursing*, *licking/grooming pups*, *sniffing nest*, *carrying pup* while BALB/c mothers performed more *blanket nursing*, *eating*, *moving pups*, *rearing*, *digging*, and *sleeping*. In order to evaluate if the strain effect observed for these variables was influenced by the size and sex ratio of each litter ANCOVAs of *arched-back nursing*, *licking/grooming pups*, *sniffing nest*, *carrying pup*, *blanket nursing*, *eating*, *moving pups*, *rearing*, and *digging* behaviors were performed. These analyses showed that the strain effect above described was not influenced by the size and the sex ratio of each litter.

**Table 1:** Frequency of observed maternal behaviors in inbred and reciprocal hybrid mothers.

*BEHAVIOR*	C57BL/6	BALB/c	F (1,50)	P-value	B6xC	CxB6	F(1,75)	P-value
*Arched-back nursing*	**0.312±0.025**	**0.158±0.021**	**25.049**	**0.001**	0.363±0.021	0.416±0.020	3.392	n.s.
*Blanket nursing*	**0.270±0.028**	**0.428±0.028**	**15.763**	**0.001**	0.206±0.024	0.172±0.018	1.357	n.s.
*Licking/grooming pups*	**0.137±0.007**	**0.045±0.003**	**150.853**	**0.001**	**0.130±0.006**	**0.091±0.004**	**30.306**	**0.001**
*Self grooming (in nest)*	0.014±0.002	0.019±0.003	1.731	n.s.	0.022±0.002	0.026±0.002	2.498	n.s.
*Sniffing nest*	**0.029±0.003**	**0.012±0.001**	**31.594**	**0.001**	0.007±0.001	0.009±0.001	2.672	n.s.
*Self grooming (out of nest)*	0.029±0.007	0.026±0.003	0.128	n.s.	0.031±0.002	0.034±0.003	0.698	n.s.
*Sniffing cage*	0.052±0.004	0.067±0.006	3.853	n.s	0.071±0.004	0.073±0.004	0.090	n.s.
*Eating*	**0.079±0.005**	**0.119±0.007**	**21.747**	**0.001**	0.085±0.005	0.083±0.004	0.088	n.s.
*Carrying pup*	**0.001±0.000**	**0.000±0.000**	**6.683**	**0.013**	0.000±0.000	0.000±0.000	0.947	n.s.
*Moving pups*	**0.002±0.000**	**0.006±0.001**	**17.920**	**0.001**	0.001±0.000	0.002±0.000	0.383	n.s.
*Nest Bulding*	0.012±0.002	0.010±0.001	0.506	n.s.	0.007±0.001	0.006±0.001	0.187	n.s.
*Sniffing pups*	0.010±0.001	0.012±0.001	0.786	n.s.	**0.005±0.001**	**0.010±0.001**	**17.214**	**0.001**
*Drinking*	0.015±0.002	0.021±0.002	4.021	n.s.	0.022±0.001	0.020±0.002	0.832	n.s.
*Rearing*	**0.006±0.001**	**0.012±0.001**	**9.479**	**0.003**	0.006±0.001	0.007±0.001	1.193	n.s.
*Digging*	**0.016±0.002**	**0.039±0.005**	**15.640**	**0.001**	0.014±0.002	0.015±0.002	0.154	n.s.
*Carrying tail*	0.006±0.001	0.008±0.002	0.778	n.s.	0.002±0.000	0.003±0.001	1.133	n.s.
*Climbing*	0.008±0.002	0.006±0.002	0.884	n.s.	0.006±0.001	0.003±0.001	3.092	n.s.
*Arched-back nursing (<half litter)*	0.000±0.000	0.001±0.000	1.207	n.s.	0.001±0.000	0.000±0.000	2.243	n.s.
*Licking/grooming pups (<half litter)*	0.000±0.000	0.001±0.000	1.859	n.s.	0.000±0.000	0.000±0.000	0.057	n.s.
*Blanket nursing (<half litter)*	0.000±0.000	0.004±0.004	1.337	n.s.	0.001±0.000	0.001±0.001	0.095	n.s.
*Sleeping*	**0.000 ± 0.000**	**0.011 ± 0.038**	**7.989**	**0.007**	0.017±0.003	0.026±0.004	2.683	n.s.

Mean frequencies (± SEM) of an ethogram of twenty-one observed maternal behaviors for C57BL/6 vs. BALB/c (**left**) and B6xC vs. CxB6 (**right**) mothers. Behaviors were scored once each minute for two non-contiguous hours each day from postnatal day 1 to 7. Statistical significance was calculated by univariate ANOVA and significant differences (P<0.05) are indicated in bold.

For reciprocal hybrid mothers, MANOVA of behavioral variables revealed a significant main effect of strain (λ = 0.457, F [20, 56] = 3.3, P = 0.001). Univariate results (see **Table 1**) showed that C57BL/6×BALB/c (B6xC) mothers performed more *licking/grooming pups* while BALB/c×C57BL/6 (CxB6) mothers performed more *sniffing pups*. ANCOVAs of *licking/grooming pups* and *sniffing pups* behaviors showed that the strain effect observed in reciprocal hybrid mothers was independent from the size and the sex ratio of each litter.

For inbred mothers, repeated measures ANOVA revealed a significant effect of strain for *licking/grooming pups* (F [1, 20] = 50.5, P = 0.001; **Figure 1B**) and *arched-back nursing* (F [1, 20] = 14.8, P = 0.001; **Figure 1C**) with C57BL/6 mothers spending more time licking and grooming pups and performing arched-back nursing than BALB/c mothers. A significant effect of day was observed for *licking/grooming pups* (F [12, 240] = 3.73, P = 0.000; **Figure 1B**), and for *arched-back nursing* (F [20, 240] = 3.75, P = 0.000; **Figure 1C**) with these behaviors decreasing significantly during the postnatal period. For F1 hybrid mothers, repeated measures ANOVA revealed a significant effect of strain on *licking/grooming pups* (F [1, 18] = 7.91, P = 0.0115; **Figure 1E**), but not *arched-back nursing* (F [1, 18] = 0.63, P = 0.438; **Figure 1F**) with B6xC mothers performing more *licking/grooming pups* than CxB6 mothers. A significant effect of day on *licking/grooming pups* (F [12, 216] = 17.2, P = 0.001, **Figure 1E**) and *arched-back nursing* (F [12, 216] = 14.7, P = 0.001, **Figure 1F**) was observed with *licking/grooming pups* and *arched-back nursing* decreasing significantly during the postnatal period. Together, these findings confirm previous reports of increased maternal care in C57BL/6 mothers compared to BALB/c mothers and demonstrate that only a small fraction of these differences are passed on to their reciprocal hybrid female offspring [3], [6], [10], [15], [16].

### Building a robust hidden Markov model of behavioral sequences

**Figure 2:**
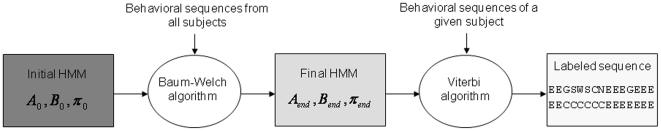
Hidden Markov model labeling of maternal behavior. An HMM is characterized by a state transition probability matrix *A*, an observation probability matrix *B*, and a state probability matrix *π*. The Baum-Welch algorithm is used to calculate a final HMM from an initial HMM (with user-defined, estimated probabilities) by maximizing the likelihood of emitting the observed behavioral sequence data. The final HMM is then used to label the behavioral sequence of each subject by application of the Viterbi algorithm. A statistical assessment of the frequency, duration, composition, and transitions between these labels can then be used to document strain differences in behavior.

Based on our previous observations [10] we specified seven HMM states: blanket nursing (BLN), arched-back nursing (ABN), licking/grooming pups (LG), grooming (GRO), activity (ACT), eating (EAT), and sleeping (SLP). Initial conditions for the HMM were chosen consistent with our previous data (see **Table S1, S2**, and **S3**) [10] and a single final HMM, named MatHMM, was derived by application of the Baum-Welch algorithm to maternal behavior data from mothers of all four strains (N_total_ = 127) during the first postnatal week (**Figure 2**).

We next tested whether our model MatHMM was sensitive to our choice of initial HMM architecture and initial model parameters. We show that, when the log-likelihood of the data converges to a plateau of about −140000, which is the case for 11 out of 100 random initial models (**Figure S2**), all states of MatHMM, except GRO, are defined by the same probabilities of behavioral variables (**Figures S3, S4, S5**). This robustness to initial model settings is also true of state transition probabilities and initial state probabilities that do not vary much between MatHMM and the best randomized models (data not shown). Our model MatHMM with seven states fits the data better than MatHMM-derived models in which states ABN [resp. LG] and BLN [resp. GRO] were merged (see **Table S4**). In addition, we found that adding an eighth random state to MatHMM only leads to a small decrease of the BIC score (see **Table S4**), and that this modification does not change the structure of the final HMM (data not shown).

### HMM labeling of maternal behavior states

To describe maternal behavior in more detail we used MatHMM to identify behavioral states and quantify their frequency, composition, and transitions. MatHMM (see **Table S5, S6** and **S7**) was then used to label sequences of observed behaviors for each subject by applying the Viterbi algorithm (**Figure 2**). In this way, each observation in a sequence of maternal behavior was assigned an HMM state.

### HMM analysis of maternal behavior in C57BL/6 mice

**Figure 3:**
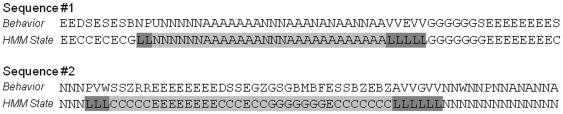
Labeling of observed behavioral sequences by HMM. The HMM formalism takes into account both the frequency and order of a series of observed behaviors to identify and label behavioral states. Examples of HMM state labeling for representative sequences of maternal behavior take from two C57BL/6 mothers (60 observations per hour). Note how bouts of nursing (A and N states) typically start and end with licking and grooming of pups (L). Also, while nursing states (A, N, L) generally last for over ten minutes, activity states (C and E) are much more brief. Observed behaviors: N = blanket nursing, R = rearing, M = moving pups, G = self-grooming in nest, Z = digging, W = sniffing nest, E = eating, D = drinking, A = arched-back nursing, U = sniffing pups, P = licking/grooming pups, S = sniffing cage, B = nest building, and V = self-grooming out of nest; HMM states: C = activity, E = eating, L = grooming pups, G = self-grooming, A = arched-back nursing, N = blanket nursing.

Initially we examined in detail HMM labelling of maternal behavior in the C57BL/6 strain. For illustration, several representative sequences of maternal behavior are shown with HMM state labelling apposed to observed behavior (**Figure 3**). Two features were apparent when comparing observed behavior with HMM states. First, HMM states were dominated by one behavior but tolerated occasional diversions to other related behaviors (e.g. *self-grooming* during EAT, *blanket nursing* during ABN). Second, a precursory analysis of the sequence of HMM states indicated a stereotypic order, with nursing bouts, for example, composed of relatively persistent stretches of ABN and BLN, and consistently beginning and ending with the LG state. On fewer occasions the GRO state intervened between nest and non-nest behavior.

An analysis of the composition of HMM states confirmed the first observation. In the C57BL/6 strain, the BLN and ABN states were exclusively composed of maternal care-related behaviors with *blanket nursing* (76%) and *arched-back nursing* (79%) dominating, respectively (see **Table S8**). The LG state was dominated by *licking/grooming pups* (66%), but was occasionally interrupted by *self-grooming* (10%), *arched-back nursing* (8.0%), and *blanket nursing* (5.2%). The GRO state appeared to be the most diverse, with contributions from both nest and non-nest behaviors: *self-grooming out of nest* (34%), *sniffing nest* (21%), *nest building* (12%), *licking/grooming pups* (8.9%), and *sniffing cage* (8.1%). The ACT state, although dominated by *sniffing cage* (37%), *digging* (13%), and *drinking* (12%), encompassed a wide range of active behaviors. Finally, the EAT state was the most monotonous, being composed almost entirely of *eating* (93%).

**Figure 4 pone-0014753-g004:**
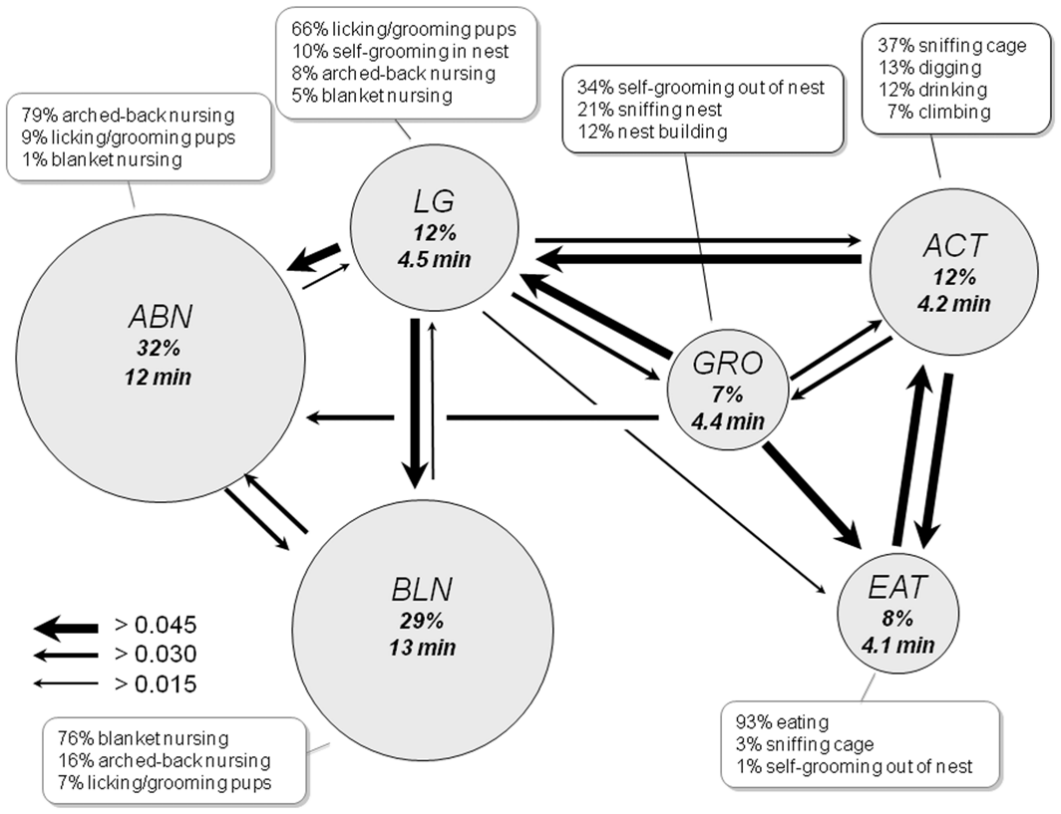
Maternal behavior strategy of C57BL/6 mothers. Graphical representation of composition, duration, frequency, and transition probabilities of HMM states for C57BL/6 mothers. Most states are composed of a single dominant behavior and multiple minor behaviors. State frequency and mean duration are indicated in site each circle. The area of each circle is proportional to state frequency. Arrows indicate absolute transition probabilities between states (transitions/minute).

In addition to precisely clustering behaviors, HMM analysis also provided information on preferred behavioral transitions between states. A graphical representation of the frequency, duration, and transition probabilities of HMM states provided a summary of the highly stereotyped behavioral patterns of C57BL/6 mothers (**Figure 4**). Nursing behavior was dominated by persistent (∼12–13 min) bouts of the ABN and BLN states which were equally favoured as initial nursing states. The major entry and exit point for nursing was a relatively short duration (∼4 min) LG state. Non-nest behavior was characterized by a rapid interchange between ACT and EAT states (∼4 min each), with ACT being the major entry and exit point for nest behavior. The GRO state served as an intermediate between nest and non-nest behavior.

### HMM analysis of maternal behavior in BALB/c mice

**Figure 5:**
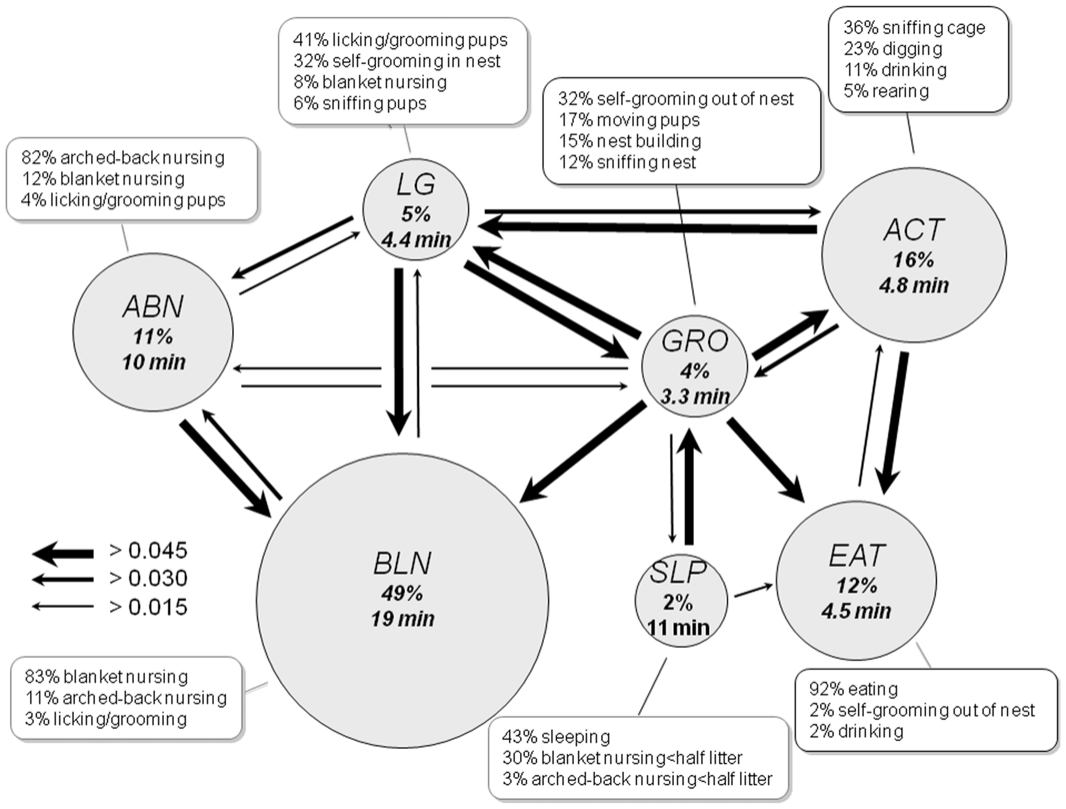
Maternal behavior strategy of BALB/c mothers. Graphical representation of composition, duration, frequency, and transition probabilities of HMM states for BALB/c mothers. Most states are composed of a single dominant behavior and multiple minor behaviors. State frequency and mean duration are indicated in site each circle. The area of each circle is proportional to state frequency. Arrows indicate absolute transition probabilities between states (transitions/minute).

Although the general structure of behavioral states in the BALB/c strain was grossly similar to that seen in the C57BL/6 strain, significant differences in HMM state frequency, composition, and transition probabilities were apparent (**Figure 5**; for detailed values and statistical significance, see **Tables S8, S9**, and **S10**). As expected, BALB/c mothers spent significantly less time in the ABN (11% vs. 32%) and LG (4.9% vs. 12%) states and significantly more time in the BLN (49% vs. 29%), ACT (16% vs. 12%), and EAT (12% vs. 8.3%) states than C57BL/6 mothers, reflecting a more self-oriented, non-nest behavioral style in this strain. Interestingly, while sleeping outside the nest (SLP) was rare in C57BL/6 mothers (0.002%), it was relatively common in BALB/c mothers (1.6%) a feature that reflected the tendency of C57BL/6 mice to sleep while nursing.

Several significant differences in HMM state composition were also seen (**Table S8**). In the BLN state, *blanket nursing* was less frequently interrupted by *arched-back nursing* (11% vs. 16%) and *licking/grooming pups* (3% vs. 7%) in BALB/c vs. C57BL/6 mothers. In the ABN state, *arched-back nursing* was less frequently interspersed with *licking/grooming pups* (4% vs. 9%) in BALB/c vs. C57BL/6 mothers. Similarly, in the LG state BALB/c mothers showed more *self-grooming* (32% vs. 10%) and less *arched-back nursing* (5% vs. 8%) than C57BL/6 mothers. Moreover, within the LG state BALB/c mothers appeared to replace *licking/grooming pups* (41% vs. 66%) with *sniffing pups* (5.9% vs. 2.4%) consistent with a less transitive maternal nurturing style.

A comparison of state transition probabilities revealed a strikingly different pattern of behavioral sequences in the two strains (**Table S10**). In BALB/c mothers the ABN state more frequently deteriorated into BLN, contributing to an increased frequency and duration of this state. In BALB/c mice the GRO state replaced the LG state as the intermediate state between nursing and non-nest behavior, with frequent transitions from this state to BLN without intervening LG. This change explained why the LG state was less frequent in BALB/c mothers, despite showing a similar duration. Finally, the EAT state was more frequent and persistent in BALB/c mice due to reduced transitions to ACT.

### HMM analysis of maternal behavior in B6xC and CxB6 mice

Overall the structure of HMM states in reciprocal hybrid mice was more similar to C57BL/6 mothers (**Figure S6, S7**; for detailed values and statistical significance, see **Tables S11, S12**, and **S13**). In both reciprocal hybrid strains the LG state served as the principal pathway in and out of nursing, while GRO typically intervened between in and out of nest behavior. However, even more than in C57BL/6 mice, nursing behavior in reciprocal hybrid mothers was dominated by ABN at the expense of BLN showing a more active nursing style in the hybrid mothers despite intermediate levels of time spent in the nest (**Figure 1D** **, S6, S7**).

As expected, differences in maternal behavior states between reciprocal hybrid mothers were highly restricted. Consistent with the maternal behavior of their mothers, B6xC mothers spent significantly more time in the LG state and less time in the GRO and SLP states than CxB6 mothers (see **Table S12** and **Figure S6, S7**). CxB6 mothers showed an increase in *self-grooming* in the LG state compared to B6xC mothers (27% vs. 17%) consistent with the more self-oriented style of the BALB/c pedigree (see **Table S11**). No significant differences in state transition probabilities between F1 hybrid mothers were observed.

## Discussion

We have provided a detailed description of maternal behavior in C57BL/6 and BALB/c inbred mice and their reciprocal F1 hybrid female offspring. We carried out observations of maternal behavior during the first three weeks of postnatal development using an ethogram of twenty-one behaviors. Analysis of the frequencies of these behaviors confirmed and extended previous findings demonstrating large differences in maternal behavior repertoire between inbred strains and the transmission of a restricted subset of these behaviors from the mothers to their female offspring. Subsequently, we subjected sequences of maternal behavior to HMM analysis to extract and describe maternal behavioral states. An analysis and comparison of the frequency, composition, and transitions of behavioral states provided a global assessment of maternal behavior in laboratory mice and revealed key differences in behavioral strategy between mouse pedigrees.

Our initial analysis of frequencies of observed maternal behaviors (**Table 1**) was consistent with previous reports demonstrating increased maternal *licking and grooming of pups* and *arched back nursing* in C57BL/6 mice when compared to BALB/c mice [4]–[6], [10], [11]. C57BL/6 mothers also spent more time in the nest, and performed more *sniffing nest* and *carrying pup* while BALB/c mothers spent more time in *blanket nursing*, *eating*, *moving pups*, *rearing*, *digging*, and *sleeping* (**Figure 1A–C** and **Table 1**). These data suggest that C57BL/6 mothers favoured pup-oriented behavior while BALB/c mothers favoured exploration of their environment and self-oriented behavior. Finally, C57BL/6 mothers showed more persistent maternal care, continuing to provide high levels of nursing behavior throughout postnatal period, while nursing behaviors of BALB/c mothers declined sharply after postnatal day 10 (**Figure 1B–C**).

Strikingly, differences in frequencies of behavior between reciprocal hybrid strains were restricted to *licking/grooming pups* and *sniffing pups*, with the former greater in B6xC and the latter greater in CxB6 mothers during all postnatal period. These results are different from the ones obtained from previous paper [15] where no overall strain differences were observed between these reciprocal hybrid mothers, but only a drastic drop of the maternal care in CxB6 at postnatal day 10. Our findings are instead in line with previous reported data [3], [10], [16], [26] suggesting the existence of non-genetic inheritance of licking and grooming behavior within these pedigrees. However it is not possible to completely rule out that imprinted genes partially contribute to the differences we observe between reciprocal hybrid strains. Our data also reveal that the transmission from the mothers to their female offspring of maternal behavior is limited to subsets of the maternal behavior repertoire with special adaptive function. It may be, for example, that this mode of inheritance helps the mother to ensure consistent levels of offspring care and foraging over generations in a way that is resistant to the introduction of genetic variation into her pedigree. This hypothesis is supported by the finding that offspring of B6xC and CxB6 reciprocal hybrid mothers show significant differences in anxiety and exploratory behaviour [10], [15], [16].

HMM analysis allowed us to go beyond a simple assessment of frequency to assess temporal associations and transitions between behaviors. The HMM formalism has been extensively used to analyse sequences of speech and gestures [17], [21] and also the analysis of protein and DNA sequences [18], [20]. Markovian models have been rarely used to analyse behavioral mouse data [22], [23]. Hidden Markov models are particularly powerful at identifying events that are clustered in time and the precise transition points between these clusters, or states. Such an analysis is particularly adapted to describe behavioral sequences in which the animal shifts between modes of activity that are dominated by one behavior but frequently interrupted by minor behaviors. By clustering frequently associated behaviors precisely determining their transitions, HMM states give a more accurate description of behavioral patterns.

Behavior of C57BL/6 mothers was dominated by interchanges between the arched-back nursing (ABN) and licking and grooming (LG) states, with the latter serving to anchor the former and act as a punctuation between nest and non-nest behavior (see also **Figure 1**). Non-nest behavior involved rapid interchanges between activity (ACT) and eating (EAT). Interestingly, drinking was primarily assigned to the ACT state, suggesting that this behavior preferentially interrupted exploratory cage activity rather than consumptive behavior. Finally the grooming (GRO) state was unique in its heterogeneity and appeared to serve as a brief transition state between nest and non-nest behavior. It is also the state which is less robust to a variation of initial model parameter settings (**Figure S5**).

Several features distinguished the structure of maternal behavior in BALB/c and C57BL/6 mothers.

Most strikingly, in BALB/c mothers LG did not serve as a central transition state between nursing and non-nursing behavior (**Figure 5**). Instead, direct transitions between the GRO state and nursing states were frequent and when coupled with an increase in the frequency and duration of the BLN state at the expense of the ABN state, contributed to a looser and less stereotyped nursing behavior in this strain. Finally, while BALB/c mothers spent considerable time sleeping outside of the nest (SLP), C57BL/6 mothers never showed this behavior, instead resting in the nest (generally as part of BLN). Overall the structure of maternal behavior as evidenced by the HMM suggested different maternal styles in the two strains, with C57BL/6 mothers being more pup-oriented and BALB/c mothers being more self and environment-oriented. It remains possible that at least some of the behavioral differences observed between C57BL/6 and BALB/c mothers derive from differences in behavior of their offspring. However, although we did not directly measure pup ultrasound vocalization, for example, that fact that offspring of our C57BL/6 and BALB/c mothers were genetically similar reciprocal hybrids (CxB6 and B6xC, respectively) is likely to have minimized such differences. Finally, we speculate that the differences in maternal behavior between C57BL/6 and BALB/c mothers reflect components of adaptive strategies found in wild mice that have become fixed in laboratory inbred strains as part of their mosaic genetic origin. It may be, for example, that changes in foraging demand selected for a more environment or more pup-oriented behavioral strategy.

Our HMM analysis showed that reciprocal hybrid strains, on the other hand, behaved similarly to each other as suggested by our initial analysis of observed variables but differed from both of their parental strains (**Figure S6, S7**). In general, reciprocal hybrids showed more stable nursing behavior, with longer and more frequent ABN punctuated (as in C57BL/6) by LG. Interestingly, like BALB/c mothers, reciprocal hybrids spend a significant amount of time sleeping out of the nest (SLP), underscoring the unusual nature of the nest-sleeping seen in C57BL/6 mothers and suggesting that it might be part of their pup-oriented maternal strategy. An assessment of the frequency, duration, composition, and transitions between HMM states exposed several features of maternal behavior not revealed by simple frequency analysis of observed behaviour. For example, comparing the p-values obtained by MANOVA of the raw variables (see **Table 1**) with the same values obtained by standard t-test of HMM state frequencies (see **Table S9, 12**) we can conclude that this second analysis produces more statistically significant differences than the first one. Additionally a specific feature of the HMM analysis is the possibility to gather, from single observed behaviors, the entire behavioral sequence of a mother during one hour of observation (for example see **Figure 3** **–** [Fig pone-0014753-g004] **5**). This kind of analysis is impossible using classical statistical methods (e.g ANOVA, MANOVA, Principal Component Analysis, etc.) which require a pooling of temporal sequences into frequencies. HMM analysis instead not only allows to describe behavioral sequences/states (clustering frequently associated behaviors), but also to perform more sophisticated analyses on the frequencies of behaviors within HMM states and frequencies of transitions between HMM states (see **Table S8, S10, S11, and S13**), to also detect even subtle differences between individuals. For instance, a MANOVA analysis on raw behavioural variables did not allow to distinguish between reciprocal hybrid mothers (see **Table 1**), whereas our HMM analysis did (see **Table S11, S12**).

We made several assumptions during our HMM analysis that may have biased our results. First, for simplicity we opted to train a single HMM, MatHMM, on behavioral sequences from all mothers, assuming that the gross structure of maternal behavior was similar between strains. Second, our choice of seven HMM states, although based on extensive earlier experience in scoring maternal behavior in these strains [10], [16] clearly forced the clustering of behavior into a limited number of states. We tested the validity of these assumptions and found that MatHMM was a better model than more simple models where ABN [resp. LG] and BLN [resp.GRO] states were merged (see **Table S4**), indicating that the presence of all these states are necessary to capture the specificities of BALB/c and C57BL/6 mouse behavioral strategies. Tests done on more complex models where a random state was added to MatHMM suggested that our seven-state model is sufficiently complex to adequately model the heterogeneity in our set of sequences (see **Table S4**). Furthermore our behavioural profile analysis on randomized models (**Figures S2, S3, S4, and S5**) strongly indicates that most states from our model MatHMM are not sensitive to variations in the initial parameters that were initially imposed. This suggests that LG, ABN, BLN, SLP, ACT, EAT states are likely to be good general descriptors of behavioral sequences in mouse laboratory strains, possibly reflecting distinct neurophysiological states of the animal, although as yet no data exists to support this hypothesis. In conclusion, although we cannot rule out that more complex models could better explain the behavioral data, we find that our results should not be biased by our choice of initial parameters.

In summary, we have applied the HMM formalism to extract behavioral states from sequences of observed behaviors. A comparison of HMM analysis with traditional frequency analysis underscored the power of this technique to reveal critical information about clustering among behaviors and about transitions between these behavioral states. The analysis of behavioural sequences is an important tool to reveal behavioral strategies adopted by different individuals. Up to now mouse behavioral studies have been limited to the analysis of frequencies and duration of single behavioral variables and have rarely focused their attention on the analysis of the global behavioral strategy adopted. We expect that the implementation of classical methods used to analyse mouse behavior in combination with HMM formalism will facilitate the gathering of important information on brain mechanisms underlying the construction of behavioural strategies. This may further our understanding of human pathologies where the execution of simple behaviors is intact but the use of complex behavioral sequences/strategies is compromised.

Finally, our findings provide a comprehensive description of maternal behavior in the laboratory mouse, reveal distinct maternal strategies in the C56BL/6 and BALB/c inbred strains, and show that the transmission of maternal behaviour from mother to daughter in this species is restricted to key behaviors with potential adaptive value.

## Supporting Information

Table S1 Initial HMM state transition (A0) matrix.
(0.04 MB DOC)

Table S2 Initial HMM state emission (B0) matrix.
(0.07 MB DOC)

Table S3 Initial HMM initial state probability (π0) matrix
(0.03 MB DOC)

Table S4 BIC scores for MatHMM-derived models
(0.03 MB DOC)

Table S5 Final HMM state transition (A0) matrix.
(0.04 MB DOC)

Table S6 Final HMM state emission (B0) matrix.
(0.06 MB DOC)

Table S7 Final HMM initial state probability (π0) matrix.
(0.03 MB DOC)

Table S8 Frequencies of behaviors within HMM states for inbred mothers. Significant strain differences as calculated by the binomial test with significance determined by FDR are indicated in bold.
(0.14 MB DOC)

Table S9 Frequency of HMM states in inbred mothers. Significant strain differences as calculated by the binomial test with significance determined by FDR are indicated in bold.
(0.04 MB DOC)

Table S10 Frequencies of transition between HMM states in inbred mothers. Significant strain differences as calculated by the binomial test with significance determined by FDR are indicated in bold.
(0.07 MB DOC)

Table S11 Frequencies of behaviors within HMM states in reciprocal hybrid mothers. Significant strain differences as calculated by the binomial test with significance determined by FDR are indicated in bold.
(0.16 MB DOC)

Table S12 Frequency of HMM states in reciprocal hybrid mothers. Significant strain differences as calculated by the binomial test with significance determined by FDR are indicated in bold.
(0.03 MB DOC)

Table S13 Frequencies of transition between HMM states in reciprocal hybrid mothers. Significant strain differences as calculated by the binomial test with significance determined by FDR are indicated in bold.
(0.08 MB DOC)

Figure S1 Observed maternal behavior in all mothers for four hours per day. Daily observations of maternal behavior from birth to weaning for 4 hours per day showed that the highest levels of (A) arched-back nursing time in nest, (B) blanket nursing, and (C) licking/grooming pups were observable during the first two hours of the daily observation (C57BL/6, N = 26; BALB/c, N = 26; B6xC, N = 36; CxB6, N = 39; * P<0.05, ** P<0.01, *** P<0.001).
(0.40 MB TIF)

Figure S2 Distribution of final log-likelihood of data for randomized models. The histogram of log-likelihoods of data after training by Baum-Welch for 100 7-states HMMs where initial parameters (A0, B0, π0) were randomized shows that random initial models converge to a final HMM which is not significantly better (i.e. its likelihood is not significantly higher) than the model MatHMM we have used to label all behavioral sequences. Only models with a final log-likelihood in the same range as the log-likelihood of MatHMM [−141000,−143000] were considered for a more detailed analysis (Figure S3, S4, S5).
(0.43 MB TIF)

Figure S3 Behavioral profiles of ABN and BLN-like states for selected randomized HMMs. This figure compares, for any given state, the probabilities of observing behavioral variables for MatHMM and the 11 most relevant randomized HMMs (see figure S2) before (B0) and after training (Bend). To avoid overloading the graph, only behavioral variables A = arched back nursing, C = climbing, E = eating, GP = grooming pups, SE = Sniffing nest, SL = sleeping, N = blanket nursing, GN = self-grooming in nest, S = sniffing cage, A+ = arched-back nursing(<half litter), N+ = no blanket nursing (<half litter) were labeled on the x-axis and only two or three models were labeled on the z-axis (MatHMM, model #42/100, model #64/100, and model #74/100). We show that for each of the 11 final randomized models (randomized models after training) we can associate states which are nearly identical to the MatHMM ABN and BLN states.
(1.27 MB TIF)

Figure S4 Behavioral profiles of ACT, EAT and SLP-like states for selected randomized HMMs. Same as Figure S3 except comparison of behavioral profiles is conducted for ACT, EAT and SLP-like states. All three states ACT, EAT and SLP that were defined for MatHMM are found in all 11 final randomized models.
(1.18 MB TIF)

Figure S5 Behavioral profiles of LG and and GRO-like states for selected randomized HMMs. Same as Figure S3 except comparison of behavioral profiles is conducted for LG and GRO-like states. LG-like but not GRO-like states were found in the 11 final randomized models.
(1.39 MB TIF)

Figure S6 Maternal behavior strategy of B6xC mothers. Graphical representation of composition, duration, frequency, and transition probabilities of HMM states for B6xC mothers. Most states are composed of a single dominant behavior and multiple minor behaviors. State frequency and mean duration are indicated in site each circle. The area of each circle is proportional to state frequency. Arrows indicate absolute transition probabilities between states (transitions/minute).
(1.09 MB TIF)

Figure S7 Maternal behavior strategy of CxB6 mothers. Graphical representation of composition, duration, frequency, and transition probabilities of HMM states for CxB6 mothers. Most states are composed of a single dominant behavior and multiple minor behaviors. State frequency and mean duration are indicated in site each circle. The area of each circle is proportional to state frequency. Arrows indicate absolute transition probabilities between states (transitions/minute).
(1.11 MB TIF)
