# Supplementary material for: Hidden Markov Model Analysis of Maternal Behavior Patterns in Inbred and Reciprocal Hybrid Mice
Source: PLoS One. 2011 Mar 8;6(3):e14753. doi: 10.1371/journal.pone.0014753 (PMC3050935; doi:10.1371/journal.pone.0014753)
Supplement: Table S1 — Initial HMM state transition (A0) matrix. (0.04 MB DOC) [file pone.0014753.s001.doc]

| *STATE* | *STATE* | | | | | | |
| --- | --- | --- | --- | --- | --- | --- | --- |
|  | **BLN** | **ABN** | **LG** | **GRO** | **ACT** | **EAT** | **SLP** |
| **BLN** | 0.1429 | 0.1429 | 0.1429 | 0.1429 | 0.1429 | 0.1429 | 0.1429 |
| **ABN** | 0.1429 | 0.1429 | 0.1429 | 0.1429 | 0.1429 | 0.1429 | 0.1429 |
| **LG** | 0.1429 | 0.1429 | 0.1429 | 0.1429 | 0.1429 | 0.1429 | 0.1429 |
| **GRO** | 0.1429 | 0.1429 | 0.1429 | 0.1429 | 0.1429 | 0.1429 | 0.1429 |
| **EAT** | 0.1429 | 0.1429 | 0.1429 | 0.1429 | 0.1429 | 0.1429 | 0.1429 |
| **ACT** | 0.1429 | 0.1429 | 0.1429 | 0.1429 | 0.1429 | 0.1429 | 0.1429 |
| **SLP** | 0.1429 | 0.1429 | 0.1429 | 0.1429 | 0.1429 | 0.1429 | 0.1429 |

Carola et al.,Table S1
