# Supplementary material for: Hidden Markov Model Analysis of Maternal Behavior Patterns in Inbred and Reciprocal Hybrid Mice
Source: PLoS One. 2011 Mar 8;6(3):e14753. doi: 10.1371/journal.pone.0014753 (PMC3050935; doi:10.1371/journal.pone.0014753)
Supplement: Table S2 — Initial HMM state emission (B0) matrix. (0.07 MB DOC) [file pone.0014753.s002.doc]

| *BEHAVIOR* | *STATE* | | | | | | |
| --- | --- | --- | --- | --- | --- | --- | --- |
|  | **BLN** | **ABN** | **LG** | **GRO** | **ACT** | **EAT** | **SLP** |
| *Arched-back nursing* | 0.001 | 0.680 | 0.001 | 0.001 | 0.001 | 0.001 | 0.001 |
| *Blanket nursing* | 0.680 | 0.001 | 0.001 | 0.001 | 0.001 | 0.001 | 0.001 |
| *Licking/grooming pups* | 0.300 | 0.300 | 0.980 | 0.001 | 0.001 | 0.001 | 0.001 |
| *Self grooming (in nest)* | 0.001 | 0.003 | 0.001 | 0.001 | 0.001 | 0.001 | 0.001 |
| *Sniffing nest* | 0.001 | 0.001 | 0.001 | 0.327 | 0.001 | 0.001 | 0.001 |
| *Self grooming (out of nest)* | 0.001 | 0.001 | 0.001 | 0.327 | 0.001 | 0.001 | 0.001 |
| *Sniffing cage* | 0.001 | 0.001 | 0.001 | 0.001 | 0.001 | 0.001 | 0.001 |
| *Eating* | 0.001 | 0.001 | 0.001 | 0.001 | 0.141 | 0.980 | 0.001 |
| *Carrying pup* | 0.001 | 0.001 | 0.001 | 0.001 | 0.001 | 0.001 | 0.001 |
| *Moving pups* | 0.001 | 0.001 | 0.001 | 0.001 | 0.001 | 0.001 | 0.001 |
| *Nest Bulding* | 0.001 | 0.001 | 0.001 | 0.327 | 0.001 | 0.001 | 0.001 |
| *Sniffing pups* | 0.001 | 0.001 | 0.001 | 0.001 | 0.141 | 0.001 | 0.001 |
| *Drinking* | 0.001 | 0.001 | 0.001 | 0.001 | 0.141 | 0.001 | 0.001 |
| *Rearing* | 0.001 | 0.001 | 0.001 | 0.001 | 0.141 | 0.001 | 0.001 |
| *Digging* | 0.001 | 0.001 | 0.001 | 0.001 | 0.141 | 0.001 | 0.001 |
| *Carrying tail* | 0.001 | 0.001 | 0.001 | 0.001 | 0.141 | 0.001 | 0.001 |
| *Climbing* | 0.001 | 0.001 | 0.001 | 0.001 | 0.141 | 0.001 | 0.001 |
| *Arched-back nursing (<half litter)* | 0.001 | 0.001 | 0.001 | 0.001 | 0.001 | 0.001 | 0.001 |
| *Licking/grooming pups* *(<half litter)* | 0.001 | 0.001 | 0.001 | 0.001 | 0.001 | 0.001 | 0.001 |
| *Blanket nursing (<half litter)* | 0.001 | 0.001 | 0.001 | 0.001 | 0.001 | 0.001 | 0.001 |
| *Sleeping* | 0.001 | 0.001 | 0.001 | 0.001 | 0.001 | 0.001 | 0.980 |

Carola et al., Table S2
