# Supplementary material for: Hidden Markov Model Analysis of Maternal Behavior Patterns in Inbred and Reciprocal Hybrid Mice
Source: PLoS One. 2011 Mar 8;6(3):e14753. doi: 10.1371/journal.pone.0014753 (PMC3050935; doi:10.1371/journal.pone.0014753)
Supplement: Table S4 — BIC scores for MatHMM-derived models (0.03 MB DOC) [file pone.0014753.s004.doc]

Carola et al., Table S4

| **HMM (number of states)** | **log-likelihood** | **BIC** |
| --- | --- | --- |
| MatHMM (7) | -140022 | 281600 |
| matHMM with ABN and BLN merged (6) | -152892 | 307077 |
| matHMM with LG and GRO merged (6) | -141057 | 283407 |
| matHMM with ABN and BLN merged and LG and GRO merged (5) | -153949 | 308940 |
| MatHMM+1 random state (8 states) | -139649 | 281132 |
