# Supplementary material for: Hidden Markov Model Analysis of Maternal Behavior Patterns in Inbred and Reciprocal Hybrid Mice
Source: PLoS One. 2011 Mar 8;6(3):e14753. doi: 10.1371/journal.pone.0014753 (PMC3050935; doi:10.1371/journal.pone.0014753)
Supplement: Table S5 — Final HMM state transition (A0) matrix. (0.04 MB DOC) [file pone.0014753.s005.doc]

| *STATE* | *STATE* | | | | | | |
| --- | --- | --- | --- | --- | --- | --- | --- |
|  | **BLN** | **ABN** | **LG** | **GRO** | **ACT** | **EAT** | **SLP** |
| **BLN** | **0.934** | 0.032 | 0.021 | 0.007 | 0.002 | 0.002 | 0.000 |
| **ABN** | 0.029 | **0.923** | 0.026 | 0.008 | 0.002 | 0.001 | 0.001 |
| **LG** | 0.058 | 0.101 | **0.743** | 0.039 | 0.040 | 0.017 | 0.001 |
| **GRO** | 0.028 | 0.034 | 0.066 | **0.657** | 0.100 | 0.105 | 0.007 |
| **ACT** | 0.008 | 0.012 | 0.060 | 0.054 | **0.765** | 0.099 | 0.001 |
| **EAT** | 0.003 | 0.004 | 0.008 | 0.017 | 0.190 | **0.779** | 0.000 |
| **SLP** | 0.009 | 0.009 | 0.010 | 0.029 | 0.007 | 0.016 | **0.919** |

Carola et al., Table S5
