# Supplementary material for: Hidden Markov Model Analysis of Maternal Behavior Patterns in Inbred and Reciprocal Hybrid Mice
Source: PLoS One. 2011 Mar 8;6(3):e14753. doi: 10.1371/journal.pone.0014753 (PMC3050935; doi:10.1371/journal.pone.0014753)
Supplement: Table S6 — Final HMM state emission (B0) matrix. (0.06 MB DOC) [file pone.0014753.s006.doc]

| BEHAVIOR | STATE | | | | | | |
| --- | --- | --- | --- | --- | --- | --- | --- |
|  | BLN | ABN | LG | GRO | ACT | EAT | SLP |
| Arched-back nursing | 0.154 | 0.811 | 0.112 | 0.023 | 0.015 | 0.005 | 0.008 |
| Blanket nursing | 0.769 | 0.088 | 0.066 | 0.030 | 0.012 | 0.000 | 0.018 |
| Licking/grooming pups | 0.054 | 0.079 | 0.547 | 0.068 | 0.019 | 0.001 | 0.007 |
| Self grooming (in nest) | 0.003 | 0.003 | 0.185 | 0.000 | 0.002 | 0.000 | 0.001 |
| Sniffing nest | 0.004 | 0.004 | 0.029 | 0.098 | 0.019 | 0.000 | 0.004 |
| Self grooming (out of nest) | 0.002 | 0.003 | 0.005 | 0.383 | 0.045 | 0.032 | 0.012 |
| Sniffing cage | 0.003 | 0.003 | 0.006 | 0.171 | 0.421 | 0.028 | 0.018 |
| Eating | 0.002 | 0.002 | 0.003 | 0.013 | 0.014 | 0.869 | 0.001 |
| Carrying pup | 0.000 | 0.000 | 0.000 | 0.004 | 0.000 | 0.000 | 0.000 |
| Moving pups | 0.000 | 0.000 | 0.007 | 0.026 | 0.003 | 0.000 | 0.000 |
| Nest Bulding | 0.001 | 0.000 | 0.010 | 0.095 | 0.013 | 0.000 | 0.000 |
| Sniffing pups | 0.005 | 0.004 | 0.026 | 0.046 | 0.013 | 0.000 | 0.000 |
| Drinking | 0.000 | 0.000 | 0.003 | 0.002 | 0.124 | 0.031 | 0.000 |
| Rearing | 0.000 | 0.000 | 0.000 | 0.013 | 0.042 | 0.014 | 0.001 |
| Digging | 0.000 | 0.000 | 0.002 | 0.020 | 0.144 | 0.000 | 0.000 |
| Carrying tail | 0.000 | 0.000 | 0.000 | 0.004 | 0.031 | 0.000 | 0.000 |
| Climbing | 0.000 | 0.000 | 0.000 | 0.000 | 0.038 | 0.007 | 0.000 |
| Arched-back nursing (<half litter) | 0.000 | 0.000 | 0.000 | 0.000 | 0.000 | 0.000 | 0.024 |
| Licking/grooming pups (<half litter) | 0.000 | 0.000 | 0.000 | 0.000 | 0.000 | 0.000 | 0.012 |
| Blanket nursing (<half litter) | 0.000 | 0.000 | 0.000 | 0.000 | 0.000 | 0.000 | 0.086 |
| Sleeping | 0.000 | 0.000 | 0.000 | 0.002 | 0.005 | 0.001 | 0.764 |

Carola et al.,Table S6
