# Supplementary material for: Hidden Markov Model Analysis of Maternal Behavior Patterns in Inbred and Reciprocal Hybrid Mice
Source: PLoS One. 2011 Mar 8;6(3):e14753. doi: 10.1371/journal.pone.0014753 (PMC3050935; doi:10.1371/journal.pone.0014753)
Supplement: Table S7 — Final HMM initial state probability (π0) matrix. (0.03 MB DOC) [file pone.0014753.s007.doc]

| *STATE* | **BLN** | **ABN** | **LG** | **GRO** | **ACT** | **EAT** | **SLP** |
| --- | --- | --- | --- | --- | --- | --- | --- |
|  | 0.143 | 0.273 | 0.113 | 0.074 | 0.181 | 0.142 | 0.011 |

Carola et al., Table S7
