# Supplementary material for: Hidden Markov Model Analysis of Maternal Behavior Patterns in Inbred and Reciprocal Hybrid Mice
Source: PLoS One. 2011 Mar 8;6(3):e14753. doi: 10.1371/journal.pone.0014753 (PMC3050935; doi:10.1371/journal.pone.0014753)
Supplement: Table S8 — Frequencies of behaviors within HMM states for inbred mothers. Significant strain differences as calculated by the binomial test with significance determined by FDR are indicated in bold. (0.14 MB DOC) [file pone.0014753.s008.doc]

| ***STATE*** | ***BEHAVIOR*** | ***C57BL/6 (%)*** | ***BALB/c (%)*** | ***P*** |
| --- | --- | --- | --- | --- |
| **BLN** | *Blanket nursing* | **75.60** | **82.58** | **0.0001** |
|  | *Arched-back nursing* | **15.88** | **10.77** | **0.0001** |
|  | *Licking/grooming pups* | **6.53** | **3.45** | **0.0001** |
|  | *Sniffing nest* | 0.54 | 0.38 | 0.1354 |
|  | *Sniffing pups* | 0.31 | 0.53 | 0.0525 |
|  | *Sniffing cage* | 0.30 | 0.53 | 0.0348 |
|  | *Eating* | 0.28 | 0.44 | 0.1198 |
|  | *Self grooming (out of nest)* | 0.19 | 0.27 | 0.3107 |
|  | *Nest Bulding* | 0.12 | 0.13 | 0.8621 |
|  | *Self grooming (in nest)* | **0.09** | **0.52** | **0.0001** |
|  | *Drinking* | 0.07 | 0.07 | 0.9721 |
|  | *Digging* | 0.05 | 0.16 | 0.0567 |
|  | *Carrying pup* | 0.02 | 0.01 | 0.7004 |
|  | *Sleeping* | 0.02 | 0.01 | 0.7004 |
|  | *Moving pups* | 0.00 | 0.10 | 0.0000 |
|  | *Rearing* | 0.00 | 0.03 | 0.1856 |
| **ABN** | *Arched-back nursing* | **78.77** | **81.58** | **0.0043** |
|  | *Licking/grooming pups* | **8.51** | **3.74** | **0.0001** |
|  | *Blanket nursing* | 1.00 | 11.71 | 0.0222 |
|  | *Sniffing nest* | 0.89 | 0.52 | 0.0884 |
|  | *Self grooming (out of nest)* | 0.59 | 0.61 | 0.9586 |
|  | *Sniffing pups* | 0.41 | 0.65 | 0.1392 |
|  | *Sniffing cage* | 0.34 | 0.22 | 0.3643 |
|  | *Eating* | 0.13 | 0.13 | 0.9914 |
|  | *Self grooming (in nest)* | 0.13 | 0.22 | 0.3577 |
|  | *Drinking* | 0.05 | 0.04 | 0.9234 |
|  | *Nest Bulding* | 0.03 | 0.13 | 0.0982 |
|  | *Carrying tail* | 0.03 | 0.09 | 0.3035 |
|  | *Moving pups* | 0.03 | 0.17 | 0.0293 |
|  | *Rearing* | 0.03 | 0.09 | 0.3035 |
|  | *Carrying pup* | 0.02 | 0.00 | 0.5417 |
|  | *Digging* | 0.02 | 0.04 | 0.4669 |
|  | *Sleeping* | 0.02 | 0.04 | 0.4669 |
| **LG** | *Licking/grooming pups* | **66.18** | **40.89** | **0.0001** |
|  | *Self grooming (in nest)* | **10.06** | **31.17** | **0.0001** |
|  | *Arched-back nursing* | **7.97** | **5.06** | **0.0027** |
|  | *Blanket nursing* | **5.22** | **8.10** | **0.0014** |
|  | *Sniffing nest* | **4.93** | **2.73** | **0.0042** |
|  | *Sniffing pups* | **2.38** | **5.87** | **0.0001** |
|  | *Nest Bulding* | 1.00 | 1.72 | 0.0824 |
|  | *Sniffing cage* | 0.71 | 0.40 | 0.3045 |
|  | *Eating* | 0.33 | 0.20 | 0.5214 |
|  | *Moving pups* | **0.33** | **2.83** | **0.0001** |
|  | *Drinking* | 0.25 | 0.00 | 0.1153 |
|  | *Digging* | 0.21 | 0.40 | 0.3140 |
|  | *Self grooming (out of nest)* | 0.21 | 0.51 | 0.1476 |
|  | *Carrying pup* | 0.17 | 0.10 | 0.6506 |
|  | *Carrying tail* | 0.04 | 0.00 | 0.5206 |
| **GRO** | *Self grooming (out of nest)* | 33.82 | 32.00 | 0.3707 |
|  | *Sniffing nest* | **21.23** | **11.70** | **0.0001** |
|  | *Nest Bulding* | 11.79 | 15.37 | 0.0146 |
|  | *Licking/grooming pups* | **8.86** | **5.62** | **0.0048** |
|  | *Sniffing cage* | 8.13 | 7.68 | 0.7060 |
|  | *Sniffing pups* | **4.32** | **7.34** | **0.0022** |
|  | *Blanket nursing* | 3.37 | 4.81 | 0.0854 |
|  | *Arched-back nursing* | 2.49 | 1.38 | 0.0704 |
|  | *Digging* | 1.61 | 3.10 | 0.0192 |
|  | *Moving pups* | **1.46** | **8.60** | **0.0001** |
|  | *Eating* | 0.88 | 0.69 | 0.6223 |
|  | *Carrying tail* | 0.73 | 0.11 | 0.0417 |
|  | *Carrying pup* | 0.59 | 0.23 | 0.2178 |
|  | *Rearing* | 0.59 | 0.81 | 0.5393 |
|  | *Drinking* | 0.15 | 0.00 | 0.2583 |
|  | *Licking/grooming pups (<half litter)* | 0.00 | 0.11 | 0.2106 |
|  | *Sleeping* | 0.00 | 0.34 | 0.0301 |
| **ACT** | *Sniffing cage* | 36.60 | 35.91 | 0.5977 |
|  | *Digging* | **13.30** | **22.86** | **0.0001** |
|  | *Drinking* | 12.20 | 11.07 | 0.1935 |
|  | *Climbing* | **6.70** | **3.40** | **0.0001** |
|  | *Sniffing nest* | **4.61** | **1.65** | **0.0001** |
|  | *Rearing* | 4.57 | 5.44 | 0.1450 |
|  | *Carrying tail* | 4.48 | 4.87 | 0.4986 |
|  | *Licking/grooming pups* | **3.68** | **0.69** | **0.0001** |
|  | *Self grooming (out of nest)* | **3.50** | **5.14** | **0.0037** |
|  | *Arched-back nursing* | **2.31** | **0.90** | **0.0001** |
|  | *Nest Bulding* | 1.95 | 1.23 | 0.0315 |
|  | *Sniffing pups* | 1.95 | 1.59 | 0.3153 |
|  | *Blanket nursing* | 1.82 | 2.19 | 0.3294 |
|  | *Eating* | 1.29 | 1.95 | 0.0570 |
|  | *Self grooming (in nest)* | **0.31** | **0.03** | **0.0066** |
|  | *Moving pups* | 0.31 | 0.39 | 0.6219 |
|  | *Carrying pup* | 0.27 | 0.03 | 0.0145 |
|  | *Sleeping* | **0.13** | **0.63** | **0.0052** |
| **EAT** | *Eating* | 92.64 | 92.44 | 0.8049 |
|  | *Self grooming (out of nest)* | 1.48 | 1.53 | 0.9076 |
|  | *Sniffing cage* | 1.30 | 1.04 | 0.4583 |
|  | *Blanket nursing* | 1.05 | 1.25 | 0.5676 |
|  | *Drinking* | 0.99 | 1.73 | 0.0514 |
|  | *Climbing* | 0.93 | 0.32 | 0.0114 |
|  | *Rearing* | 0.87 | 1.21 | 0.2992 |
|  | *Arched-back nursing* | 0.57 | 0.24 | 0.1030 |
|  | *Self grooming (in nest)* | 0.12 | 0.08 | 0.6650 |
|  | *Licking/grooming pups* | 0.06 | 0.08 | 0.8290 |
|  | *Sleeping* | 0.00 | 0.08 | 0.2538 |
| **SLP** | *Sleeping* | 0.00 | 43.16 | 0.2530 |
|  | *Blanket nursing (<half litter)* | 0.00 | 30.39 | 0.5090 |
|  | *Arched-back nursing (<half litter)* | 0.00 | 3.34 | 0.8524 |
|  | *Blanket nursing* | 0.00 | 2.43 | 0.8746 |
|  | *Licking/grooming pups* | 0.00 | 1.51 | 0.9011 |
|  | *Arched-back nursing* | 0.00 | 0.91 | 0.9236 |
|  | *Sniffing nest* | 0.00 | 0.91 | 0.9236 |
|  | *Eating* | 0.00 | 0.61 | 0.9377 |
|  | *Licking/grooming pups (<half litter)* | 0.00 | 0.61 | 0.9378 |
|  | *Sniffing cage* | 0.00 | 0.61 | 0.9379 |
|  | *Drinking* | 0.00 | 0.30 | 0.9560 |
|  | *Self grooming (out of nest)* | 0.00 | 0.30 | 0.9560 |
|  | *Self grooming (in nest)* | 0.00 | 0.30 | 0.9560 |
|  | *Rearing* | 0.00 | 0.30 | 0.9560 |

Carola et al.,Table S8
