# Supplementary material for: Hidden Markov Model Analysis of Maternal Behavior Patterns in Inbred and Reciprocal Hybrid Mice
Source: PLoS One. 2011 Mar 8;6(3):e14753. doi: 10.1371/journal.pone.0014753 (PMC3050935; doi:10.1371/journal.pone.0014753)
Supplement: Table S9 — Frequency of HMM states in inbred mothers. Significant strain differences as calculated by the binomial test with significance determined by FDR are indicated in bold. (0.04 MB DOC) [file pone.0014753.s009.doc]

| *BEHAVIOR* | **C57BL/6 (%)** | **BALB/c (%)** | **P-value** |
| --- | --- | --- | --- |
| **BLN** | 29.37 | 48.84 | 0.00001 |
| **ABN** | 31.58 | 11.42 | 0.00001 |
| **LG** | 12.25 | 4.90 | 0.00001 |
| **GRO** | 6.99 | 4.33 | 0.00001 |
| **ACT** | 11.53 | 16.52 | 0.00001 |
| **EAT** | 8.28 | 12.35 | 0.00001 |
| **SLP** | 0.00 | 1.64 | 0.00001 |

Carola et al., Table S9
