# Supplementary material for: Hidden Markov Model Analysis of Maternal Behavior Patterns in Inbred and Reciprocal Hybrid Mice
Source: PLoS One. 2011 Mar 8;6(3):e14753. doi: 10.1371/journal.pone.0014753 (PMC3050935; doi:10.1371/journal.pone.0014753)
Supplement: Table S10 — Frequencies of transition between HMM states in inbred mothers. Significant strain differences as calculated by the binomial test with significance determined by FDR are indicated in bold. (0.07 MB DOC) [file pone.0014753.s010.doc]

| ***from STATE*** | ***to STATE*** | ***C57BL/6 (%)*** | ***BALB/c (%)*** | ***P*** |
| --- | --- | --- | --- | --- |
| **BLN** | **ABN** | 48.94 | 41.72 | 0.0530 |
|  | **LG** | 31.12 | 31.95 | 0.8104 |
|  | **GRO** | 13.83 | 15.97 | 0.4206 |
|  | **EAT** | 4.79 | 2.37 | 0.0847 |
|  | **ACT** | **1.33** | **5.62** | **0.0015** |
|  | **SLP** | **0.00** | **2.36** | **0.0027** |
| **ABN** | **BLN** | **47.07** | **60.49** | **0.0020** |
|  | **LG** | **30.24** | **16.10** | **0.0001** |
|  | **GRO** | 17.56 | 14.63 | 0.3576 |
|  | **ACT** | 3.17 | 5.37 | 0.1851 |
|  | **EAT** | 1.71 | 1.46 | 0.8216 |
|  | **SLP** | 0.00 | 1.95 | 0.0262 |
| **LG** | **ABN** | **30.69** | **11.27** | **0.0001** |
|  | **BLN** | **27.80** | **41.45** | **0.0001** |
|  | **ACT** | 19.31 | 25.45 | 0.0446 |
|  | **GRO** | 14.86 | 16.36 | 0.5777 |
|  | **EAT** | 7.34 | 5.09 | 0.2242 |
|  | **SLP** | 0.00 | 0.36 | 0.1697 |
| **GRO** | **LG** | **28.66** | **19.54** | **0.0117** |
|  | **ABN** | **19.64** | **7.66** | **0.0001** |
|  | **EAT** | 20.01 | 29.50 | 0.6959 |
|  | **ACT** | 17.92 | 16.86 | 0.7406 |
|  | **BLN** | **0.59** | **22.60** | **0.0001** |
|  | **SLP** | **0.00** | **3.83** | **0.0001** |
| **ACT** | **LG** | **30.64** | **11.65** | **0.0001** |
|  | **EAT** | **46.10** | **63.84** | **0.0001** |
|  | **GRO** | 16.18 | 15.73 | 0.8334 |
|  | **ABN** | 4.24 | 2.12 | 0.0354 |
|  | **BLN** | **2.89** | **5.90** | **0.0140** |
|  | **SLP** | 0.00 | 0.08 | 0.0470 |
| **EAT** | **ACT** | **87.95** | **94.03** | **0.0010** |
|  | **LG** | **5.13** | **1.49** | **0.0014** |
|  | **GRO** | 4.10 | 1.68 | 0.0247 |
|  | **ABN** | 2.56 | 0.93 | 0.0522 |
|  | **BLN** | 0.26 | 1.87 | 0.0256 |
|  | **SLP** | 0.00 | 0.00 | 1 |
| **SLP** | **BLN** | 0.00 | 10.34 | 0.7346 |
|  | **ABN** | 0.00 | 6.90 | 0.7857 |
|  | **LG** | 0.00 | 6.90 | 0.7857 |
|  | **GRO** | 0.00 | 55.17 | 0.2769 |
|  | **EAT** | 0.00 | 13.80 | 0.6870 |
|  | **ACT** | 0.00 | 6.90 | 0.7858 |

Carola et al., Table S10
