# Supplementary material for: Hidden Markov Model Analysis of Maternal Behavior Patterns in Inbred and Reciprocal Hybrid Mice
Source: PLoS One. 2011 Mar 8;6(3):e14753. doi: 10.1371/journal.pone.0014753 (PMC3050935; doi:10.1371/journal.pone.0014753)
Supplement: Table S11 — Frequencies of behaviors within HMM states in reciprocal hybrid mothers. Significant strain differences as calculated by the binomial test with significance determined by FDR are indicated in bold. (0.16 MB DOC) [file pone.0014753.s011.doc]

| ***STATE*** | ***BEHAVIOR*** | ***B6xC(%)*** | ***CxB6(%)*** | ***P*** |
| --- | --- | --- | --- | --- |
| **BLN** | *Blanket nursing* | 75.82 | 75.61 | 0.7934 |
|  | *Arched-back nursing* | 16.17 | 17.18 | 0.1373 |
|  | *Licking/grooming pups* | **6.48** | **4.84** | **0.0001** |
|  | *Sniffing nest* | 0.35 | 0.58 | 0.0617 |
|  | *Sniffing cage* | 0.30 | 0.24 | 0.5724 |
|  | *Sniffing pups* | **0.27** | **0.83** | **0.0001** |
|  | *Nest Bulding* | 0.18 | 0.09 | 0.1911 |
|  | *Self grooming (in nest)* | 0.11 | 0.19 | 0.2825 |
|  | *Eating* | 0.09 | 0.19 | 0.1153 |
|  | *Self grooming (out of nest)* | 0.09 | 0.04 | 0.3031 |
|  | *Drinking* | 0.04 | 0.00 | 0.1332 |
|  | *Moving pups* | 0.03 | 0.08 | 0.2420 |
|  | *Arched-back nursing (<half litter)* | 0.01 | 0.00 | 0.3848 |
|  | *Digging* | 0.01 | 0.02 | 0.8420 |
|  | *Blanket nursing (<half litter)* | 0.01 | 0.02 | 0.8420 |
|  | *Sleeping* | 0.01 | 0.02 | 0.8420 |
|  | *Carrying pup* | 0.00 | 0.04 | 0.1036 |
|  | *Rearing* | 0.00 | 0.02 | 0.2498 |
| **ABN** | *Arched-back nursing* | **81.47** | **83.93** | **0.0001** |
|  | *Licking/grooming pups* | **9.16** | **6.76** | **0.0001** |
|  | *Blanket nursing* | 7.63 | 7.11 | 0.1117 |
|  | *Self grooming (in nest)* | 0.36 | 0.28 | 0.2533 |
|  | *Sniffing cage* | 0.33 | 0.31 | 0.7997 |
|  | *Sniffing pups* | **0.27** | **0.53** | **0.0013** |
|  | *Sniffing nest* | **0.23** | **0.47** | **0.0015** |
|  | *Eating* | 0.22 | 0.15 | 0.1667 |
|  | *Self grooming (out of nest)* | 0.09 | 0.16 | 0.1057 |
|  | *Drinking* | 0.07 | 0.07 | 0.9989 |
|  | *Nest Bulding* | 0.06 | 0.10 | 0.2671 |
|  | *Rearing* | 0.04 | 0.02 | 0.3918 |
|  | *Moving pups* | 0.02 | 0.05 | 0.1305 |
|  | *Climbing* | 0.01 | 0.00 | 0.2920 |
|  | *Carrying pup* | 0.01 | 0.00 | 0.2920 |
|  | *Digging* | 0.01 | 0.07 | 0.6360 |
|  | *Blanket nursing (<half litter)* | 0.01 | 0.01 | 0.9409 |
|  | *Sleeping* | 0.01 | 0.02 | 0.3699 |
| **LG** | *Licking/grooming pups* | **66.16** | **51.82** | **0.0001** |
|  | *Self grooming (in nest)* | **17.22** | **26.61** | **0.0001** |
|  | *Arched-back nursing* | 7.17 | 8.51 | 0.0466 |
|  | *Blanket nursing* | 4.16 | 4.31 | 0.7745 |
|  | *Sniffing nest* | 1.78 | 2.19 | 0.2388 |
|  | *Sniffing pups* | **1.40** | **3.85** | **0.0001** |
|  | *Sniffing cage* | 0.49 | 0.35 | 0.3941 |
|  | *Nest Bulding* | 0.42 | 0.95 | 0.0069 |
|  | *Moving pups* | 0.38 | 0.71 | 0.0510 |
|  | *Eating* | 0.36 | 0.14 | 0.0934 |
|  | *Self grooming (out of nest)* | 0.19 | 0.22 | 0.8574 |
|  | *Drinking* | 0.11 | 0.28 | 0.1081 |
|  | *Digging* | 0.08 | 0.04 | 0.4510 |
|  | *Carrying pup* | 0.05 | 0.00 | 0.2128 |
|  | *Arched-back nursing (<half litter)* | 0.03 | 0.00 | 0.3784 |
|  | *Carrying tail* | 0.03 | 0.00 | 0.3784 |
| **GRO** | *Self grooming (out of nest)* | 53.24 | 54.60 | 0.5196 |
|  | *Sniffing cage* | 14.36 | 16.,41 | 0.1827 |
|  | *Nest Bulding* | 9.98 | 7.90 | 0.0864 |
|  | *Sniffing nest* | 6.37 | 6.42 | 0.9581 |
|  | *Sniffing pups* | 3.90 | 4.17 | 0.7483 |
|  | *Licking/grooming pups* | 3.52 | 2.69 | 0.2627 |
|  | *Moving pups* | 2.28 | 1.22 | 0.0548 |
|  | *Blanket nursing* | 1.33 | 1.13 | 0.6662 |
|  | *Digging* | 0.95 | 1.04 | 0.8299 |
|  | *Eating* | 0.86 | 0.87 | 0.9746 |
|  | *Rearing* | 0.76 | 0.69 | 0.8553 |
|  | *Carrying pup* | 0.67 | 0.35 | 0.2897 |
|  | *Arched-back nursing* | 0.57 | 1.91 | 0.0050 |
|  | *Drinking* | 0.47 | 0.17 | 0.2087 |
|  | *Carrying tail* | 0.28 | 0.09 | 0.2744 |
|  | *Sleeping* | 0.19 | 0.35 | 0.4795 |
| **ACT** | *Sniffing cage* | 48.67 | 48.31 | 0.7453 |
|  | *Digging* | **15.57** | **12.89** | **0.0007** |
|  | *Drinking* | 10.11 | 11.51 | 0.0456 |
|  | *Self grooming (out of nest)* | 7.30 | 6.63 | 0.2458 |
|  | *Climbing* | **4.10** | **2.32** | **0.0001** |
|  | *Rearing* | 6.69 | 4.62 | 0.0385 |
|  | *Licking/grooming pups* | 1.91 | 1.49 | 0.1527 |
|  | *Eating* | 1.68 | 1.83 | 0.6211 |
|  | *Arched-back nursing* | 1.53 | 1.67 | 0.6203 |
|  | *Carrying tail* | **1.45** | **2.69** | **0.0001** |
|  | *Sniffing nest* | 0.93 | 1.46 | 0.0296 |
|  | *Nest Bulding* | 0.88 | 1.41 | 0.0267 |
|  | *Sniffing pups* | 0.75 | 1.17 | 0.0557 |
|  | *Blanket nursing* | 0.68 | 0.68 | 0.9937 |
|  | *Sleeping* | 0.43 | 0.86 | 0.0158 |
|  | *Self grooming (in nest)* | 0.15 | 0.13 | 0.8138 |
|  | *Carrying pup* | 0.10 | 0.10 | 0.9550 |
|  | *Moving pups* | 0.10 | 0.21 | 0.2206 |
|  | *Arched-back nursing (<half litter)* | 0.00 | 0.03 | 0.3076 |
| **EAT** | *Eating* | 91.72 | 91.30 | 0.5817 |
|  | *Self grooming (out of nest)* | 2.82 | 2.77 | 0.9115 |
|  | *Drinking* | 1.54 | 2.26 | 0.0530 |
|  | *Sniffing cage* | 1.14 | 1.37 | 0.4510 |
|  | *Rearing* | 1.03 | 0.98 | 0.8544 |
|  | *Arched-back nursing* | 0.59 | 0.70 | 0.5969 |
|  | *Climbing* | 0.48 | 0.27 | 0.2288 |
|  | *Blanket nursing* | 0.44 | 0.23 | 0.1994 |
|  | *Sleeping* | 0.22 | 0.04 | 0.0705 |
|  | *Licking/grooming pups* | 0.04 | 0.08 | 0.5271 |
| **SLP** | *Sleeping* | 86.17 | 82.75 | 0.0813 |
|  | *Sniffing cage* | 2.53 | 1.46 | 0.1456 |
|  | *Arched-back nursing (<half litter)* | 2.36 | 2.19 | 0.8282 |
|  | *Arched-back nursing* | **1.35** | **0.12** | **0.0041** |
|  | *Self grooming (in nest)* | 1.35 | 1.22 | 0.8242 |
|  | *Licking/grooming pups* | 0.84 | 0.12 | 0.0392 |
|  | *Blanket nursing* | 0.84 | 2.19 | 0.0484 |
|  | *Rearing* | 0.17 | 0.00 | 0.2386 |
|  | *Sniffing nest* | 0.17 | 0.12 | 0.8158 |
|  | *Digging* | 0.00 | 0.12 | 0.3958 |
|  | *Self grooming (in nest)* | 0.00 | 0.12 | 0.3958 |
|  | *Licking/grooming pups (<half litter)* | **0.00** | **2.31** | **0.0001** |
|  | *Blanket nursing (<half litter)* | **0.00** | **7.29** | **0.0001** |

Carola et al., Table S11
