# Supplementary material for: Hidden Markov Model Analysis of Maternal Behavior Patterns in Inbred and Reciprocal Hybrid Mice
Source: PLoS One. 2011 Mar 8;6(3):e14753. doi: 10.1371/journal.pone.0014753 (PMC3050935; doi:10.1371/journal.pone.0014753)
Supplement: Table S12 — Frequency of HMM states in reciprocal hybrid mothers. Significant strain differences as calculated by the binomial test with significance determined by FDR are indicated in bold. (0.03 MB DOC) [file pone.0014753.s012.doc]

| *BEHAVIOR* | **B6xC (%)** | **CxB6 (%)** | **P-value** |
| --- | --- | --- | --- |
| **BLN** | 22.64 | 17.78 | 0.00001 |
| **ABN** | 38.80 | 44.82 | 0.00001 |
| **LG** | 11.72 | 9.46 | 0.00001 |
| **GRO** | 3.38 | 3.85 | 0.00185 |
| **ACT** | 12.80 | 12.79 | n.s. |
| **EAT** | 8.76 | 8.56 | n.s. |
| **SLP** | 1.90 | 2.74 | 0.00001 |

Carola et al., Table S12
