# Supplementary material for: Hidden Markov Model Analysis of Maternal Behavior Patterns in Inbred and Reciprocal Hybrid Mice
Source: PLoS One. 2011 Mar 8;6(3):e14753. doi: 10.1371/journal.pone.0014753 (PMC3050935; doi:10.1371/journal.pone.0014753)
Supplement: Table S13 — Frequencies of transition between HMM states in reciprocal hybrid mothers. Significant strain differences as calculated by the binomial test with significance determined by FDR are indicated in bold. (0.08 MB DOC) [file pone.0014753.s013.doc]

| ***from STATE*** | ***to STATE*** | ***B6xC (%)*** | ***CxB6 (%)*** | ***P*** |
| --- | --- | --- | --- | --- |
| **BLN** | **ABN** | 50.92 | 58.19 | 0.0281 |
|  | **LG** | 34.36 | 25.18 | 0.0027 |
|  | **GRO** | 8.18 | 9.97 | 0.3454 |
|  | **EAT** | 3.27 | 2.38 | 0.4182 |
|  | **SLP** | 1.84 | 2.85 | 0.3117 |
|  | **ACT** | 1.43 | 1.43 | 0.9936 |
| **ABN** | **BLN** | 41.56 | 40.09 | 0.5820 |
|  | **LG** | 41.13 | 38.74 | 0.3692 |
|  | **GRO** | 8.88 | 12.31 | 0.0350 |
|  | **ACT** | 3.32 | 4.50 | 0.2591 |
|  | **SLP** | 3.03 | 3,15 | 0.8959 |
|  | **EAT** | 2.16 | 1.20 | 0.1687 |
| **GRO** | **EAT** | 40.73 | 47.34 | 0.0816 |
|  | **ACT** | 21.28 | 16.53 | 0.1117 |
|  | **LG** | 20.97 | 18.21 | 0.3614 |
|  | **ABN** | 6.99 | 9.24 | 0.2817 |
|  | **BLN** | 5.78 | 4.48 | 0.4419 |
|  | **SLP** | 4.26 | 4.20 | 0.9721 |
| **LG** | **ABN** | 44.46 | 46.68 | 0.3832 |
|  | **BLN** | 19.14 | 15.32 | 0.0489 |
|  | **ACT** | 17.15 | 16.47 | 0.7227 |
|  | **GRO** | 10.97 | 14.88 | 0.0214 |
|  | **EAT** | 7.82 | 6.07 | 0.1810 |
|  | **SLP** | 0.47 | 0.58 | 0.7613 |
| **ACT** | **EAT** | 44.19 | 43.64 | 0.8270 |
|  | **LG** | 36.56 | 31.59 | 0.0380 |
|  | **GRO** | 10.90 | 12.45 | 0.3371 |
|  | **ABN** | 5.57 | 8.70 | 0.0154 |
|  | **BLN** | 2.42 | 2.81 | 0.6279 |
|  | **SLP** | 0.36 | 0.80 | 0.2479 |
| **EAT** | **ACT** | 91.41 | 90.49 | 0.5821 |
|  | **GRO** | 3.90 | 3.87 | 0.9882 |
|  | **LG** | 2.43 | 2.99 | 0.5511 |
|  | **ABN** | 1.13 | 1.23 | 0.8762 |
|  | **BLN** | 1.13 | 1.41 | 0.6735 |
|  | **SLP** | 0.00 | 0.00 | 1 |
| **SLP** | **GRO** | 42.31 | 33.33 | 0.3340 |
|  | **LG** | 15.38 | 7.02 | 0.1633 |
|  | **BLN** | 13.46 | 10.53 | 0.6367 |
|  | **EAT** | 11.54 | 15.79 | 0.5199 |
|  | **ABN** | 9.61 | 17.54 | 0.2301 |
|  | **ACT** | 7.69 | 15.80 | 0.1926 |

Carola et al., Table S13
