# Supplementary figures and images for: Hidden Markov Model Analysis of Maternal Behavior Patterns in Inbred and Reciprocal Hybrid Mice
Source: PLoS One. 2011 Mar 8;6(3):e14753. doi: 10.1371/journal.pone.0014753 (PMC3050935; doi:10.1371/journal.pone.0014753)

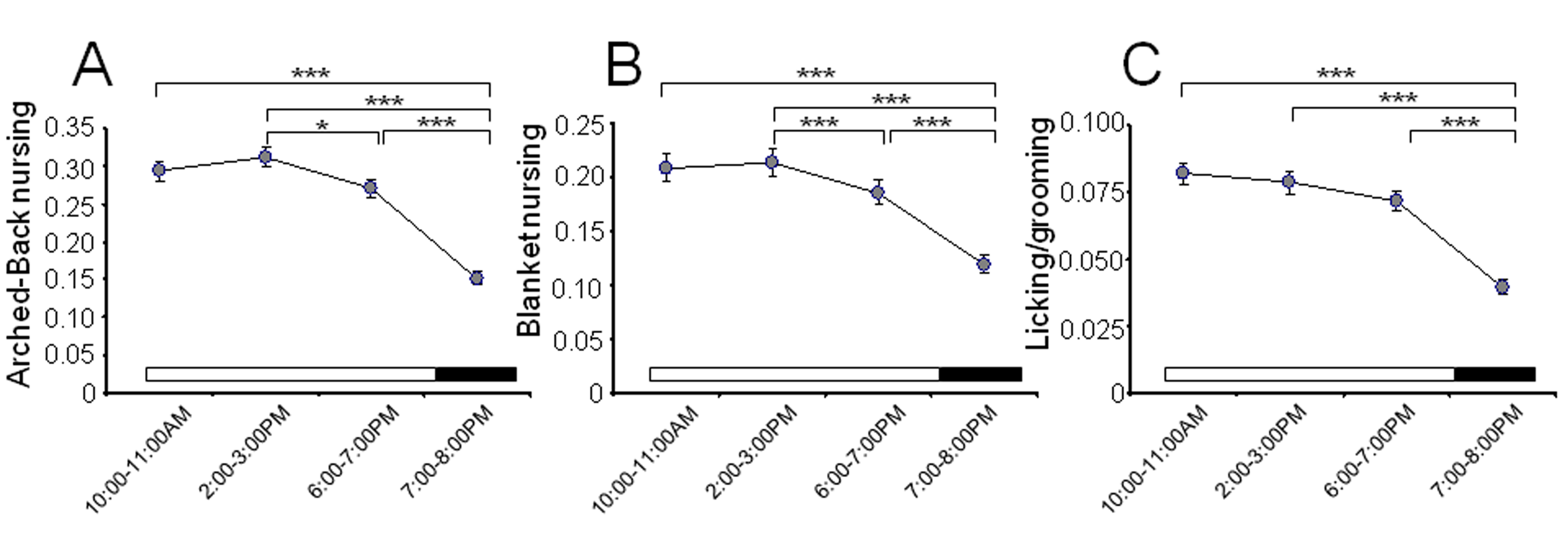

Supplement: Figure S1 — Observed maternal behavior in all mothers for four hours per day. Daily observations of maternal behavior from birth to weaning for 4 hours per day showed that the highest levels of (A) arched-back nursing time in nest, (B) blanket nursing, and (C) licking/grooming pups were observable during the first two hours of the daily observation (C57BL/6, N = 26; BALB/c, N = 26; B6xC, N = 36; CxB6, N = 39; * P<0.05, ** P<0.01, *** P<0.001). (0.40 MB TIF) [file pone.0014753.s014.tif]

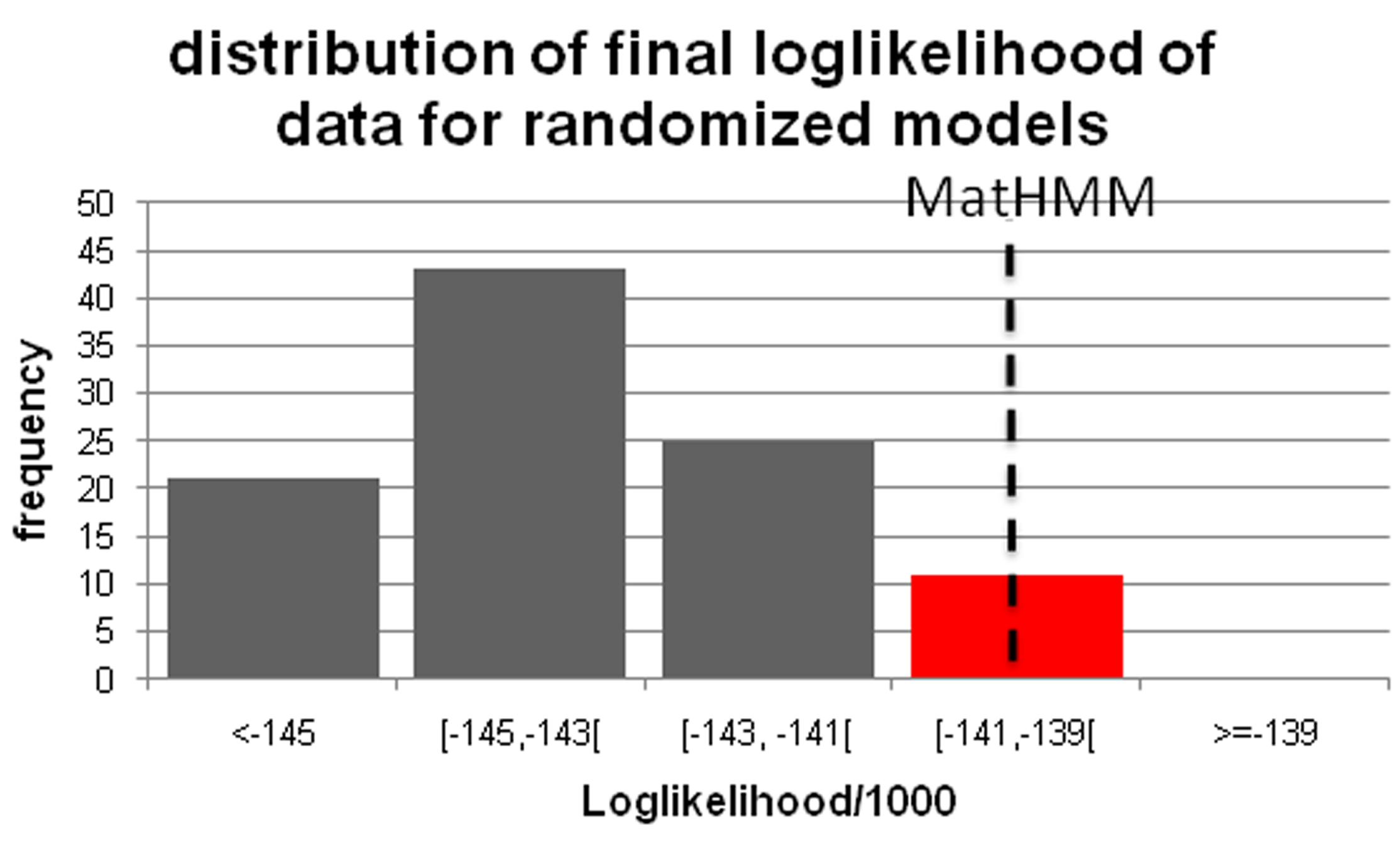

Supplement: Figure S2 — Distribution of final log-likelihood of data for randomized models. The histogram of log-likelihoods of data after training by Baum-Welch for 100 7-states HMMs where initial parameters (A0, B0, π0) were randomized shows that random initial models converge to a final HMM which is not significantly better (i.e. its likelihood is not significantly higher) than the model MatHMM we have used to label all behavioral sequences. Only models with a final log-likelihood in the same range as the log-likelihood of MatHMM [−141000,−143000] were considered for a more detailed analysis (Figure S3, S4, S5). (0.43 MB TIF) [file pone.0014753.s015.tif]

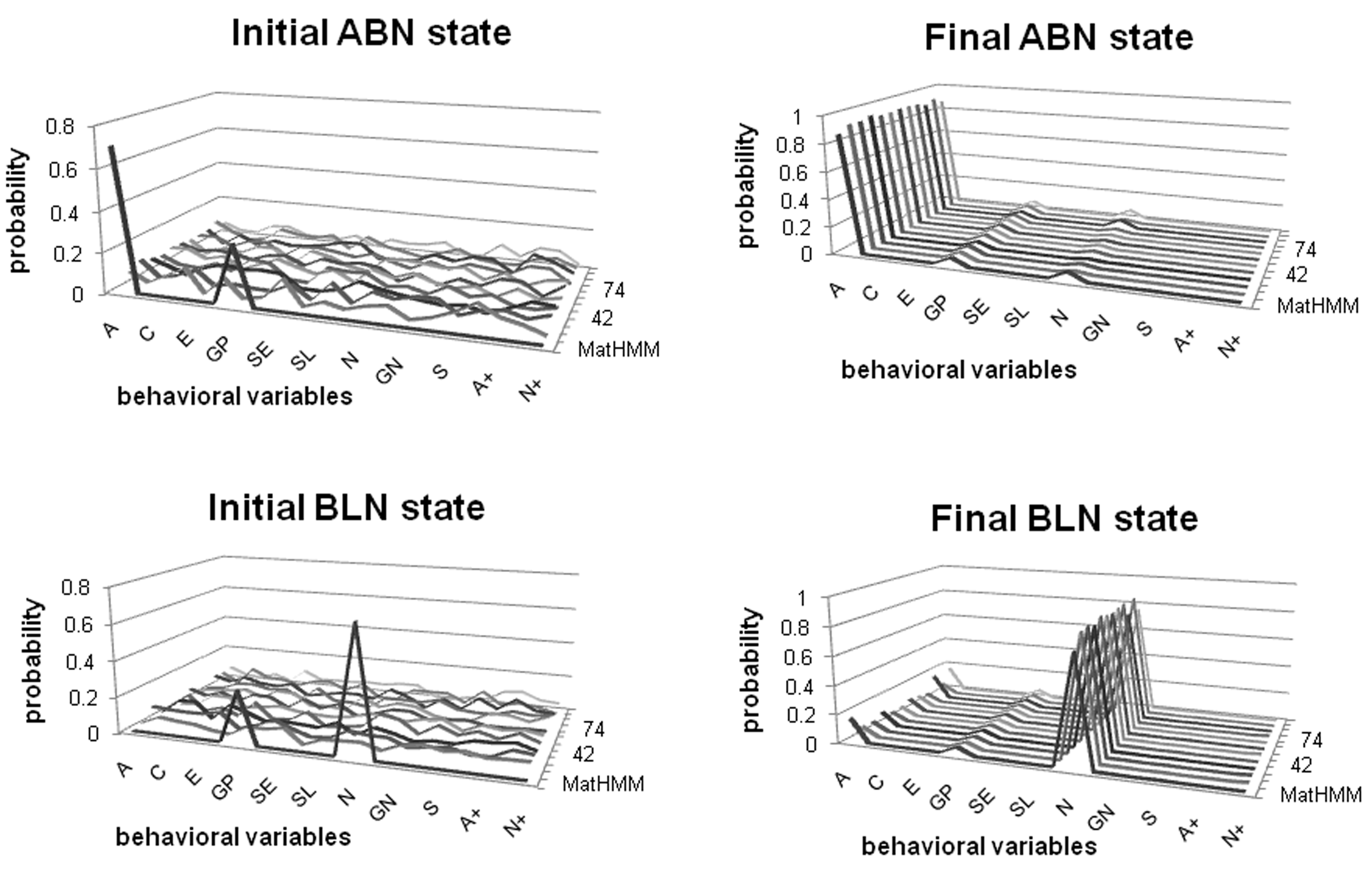

Supplement: Figure S3 — Behavioral profiles of ABN and BLN-like states for selected randomized HMMs. This figure compares, for any given state, the probabilities of observing behavioral variables for MatHMM and the 11 most relevant randomized HMMs (see figure S2) before (B0) and after training (Bend). To avoid overloading the graph, only behavioral variables A = arched back nursing, C = climbing, E = eating, GP = grooming pups, SE = Sniffing nest, SL = sleeping, N = blanket nursing, GN = self-grooming in nest, S = sniffing cage, A+ = arched-back nursing(<half litter), N+ = no blanket nursing (<half litter) were labeled on the x-axis and only two or three models were labeled on the z-axis (MatHMM, model #42/100, model #64/100, and model #74/100). We show that for each of the 11 final randomized models (randomized models after training) we can associate states which are nearly identical to the MatHMM ABN and BLN states. (1.27 MB TIF) [file pone.0014753.s016.tif]

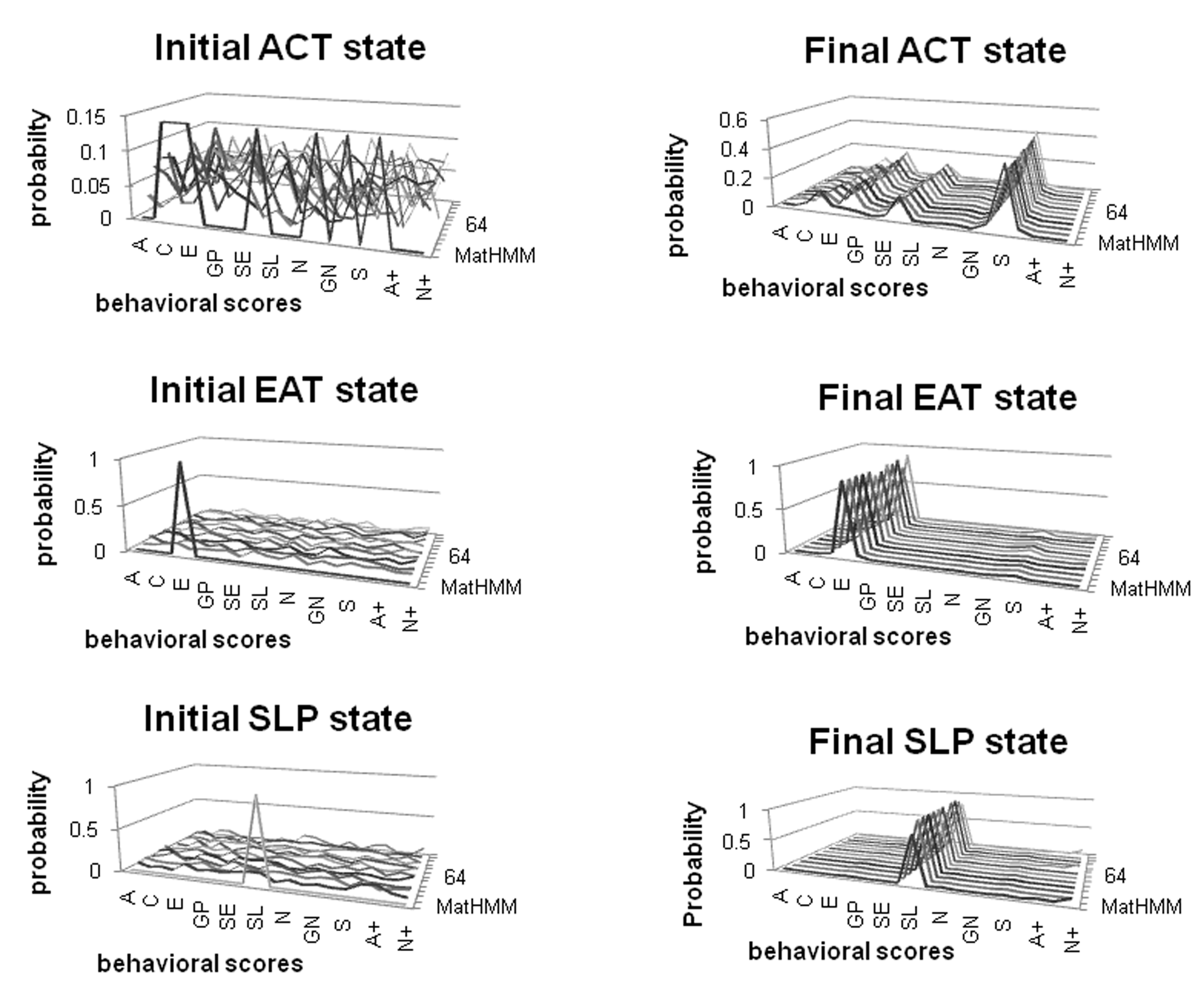

Supplement: Figure S4 — Behavioral profiles of ACT, EAT and SLP-like states for selected randomized HMMs. Same as Figure S3 except comparison of behavioral profiles is conducted for ACT, EAT and SLP-like states. All three states ACT, EAT and SLP that were defined for MatHMM are found in all 11 final randomized models. (1.18 MB TIF) [file pone.0014753.s017.tif]

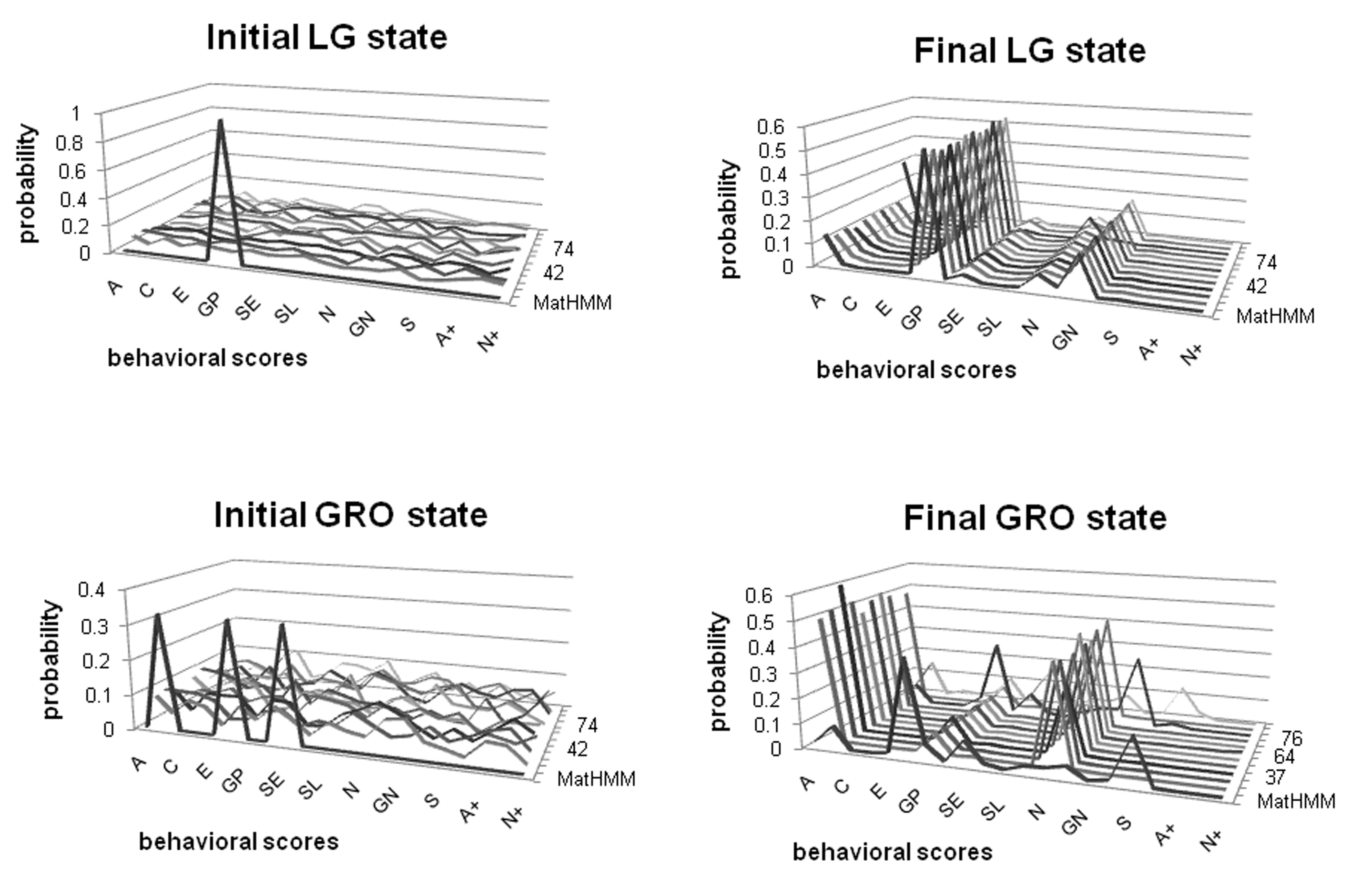

Supplement: Figure S5 — Behavioral profiles of LG and and GRO-like states for selected randomized HMMs. Same as Figure S3 except comparison of behavioral profiles is conducted for LG and GRO-like states. LG-like but not GRO-like states were found in the 11 final randomized models. (1.39 MB TIF) [file pone.0014753.s018.tif]

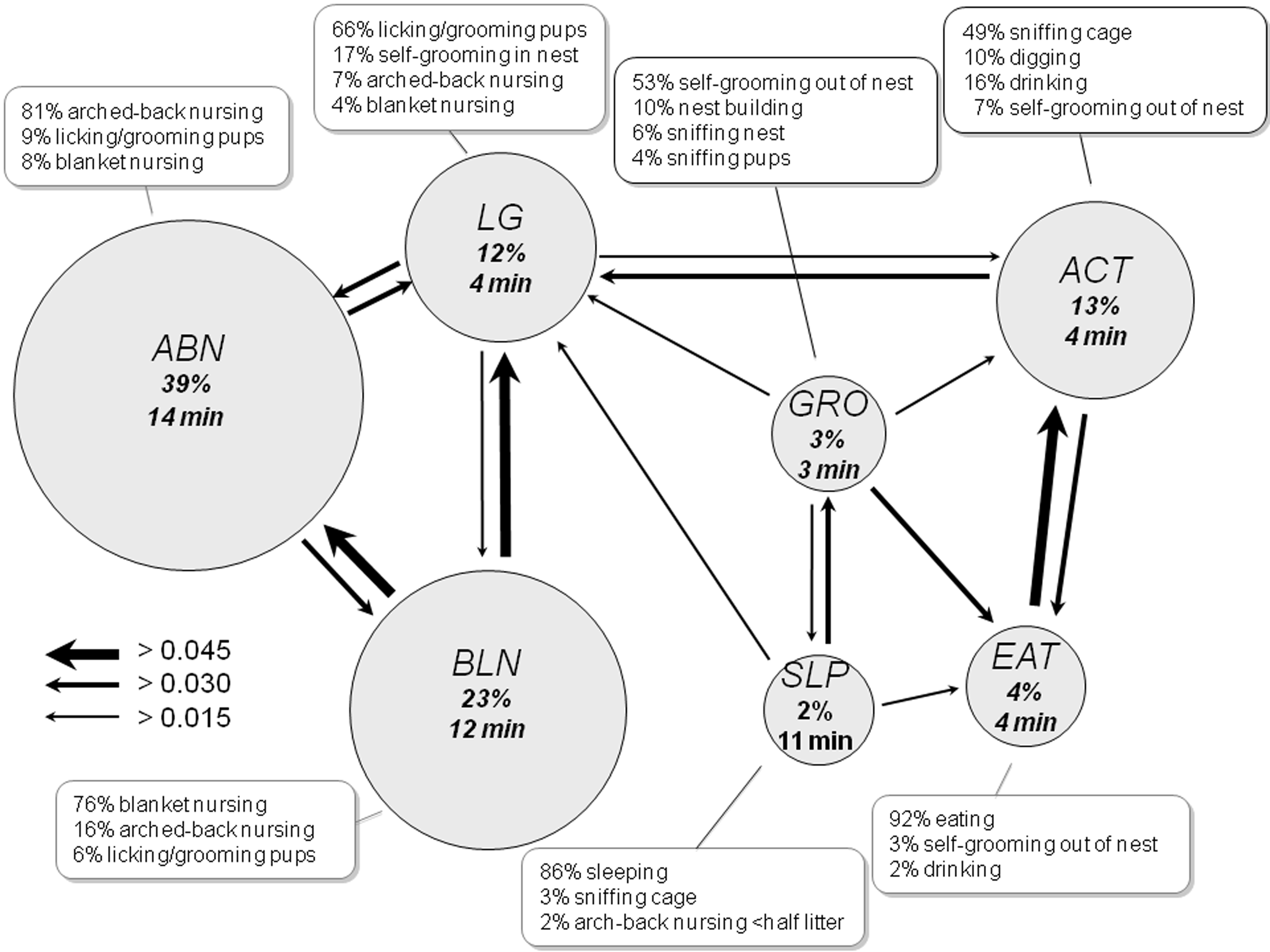

Supplement: Figure S6 — Maternal behavior strategy of B6xC mothers. Graphical representation of composition, duration, frequency, and transition probabilities of HMM states for B6xC mothers. Most states are composed of a single dominant behavior and multiple minor behaviors. State frequency and mean duration are indicated in site each circle. The area of each circle is proportional to state frequency. Arrows indicate absolute transition probabilities between states (transitions/minute). (1.09 MB TIF) [file pone.0014753.s019.tif]

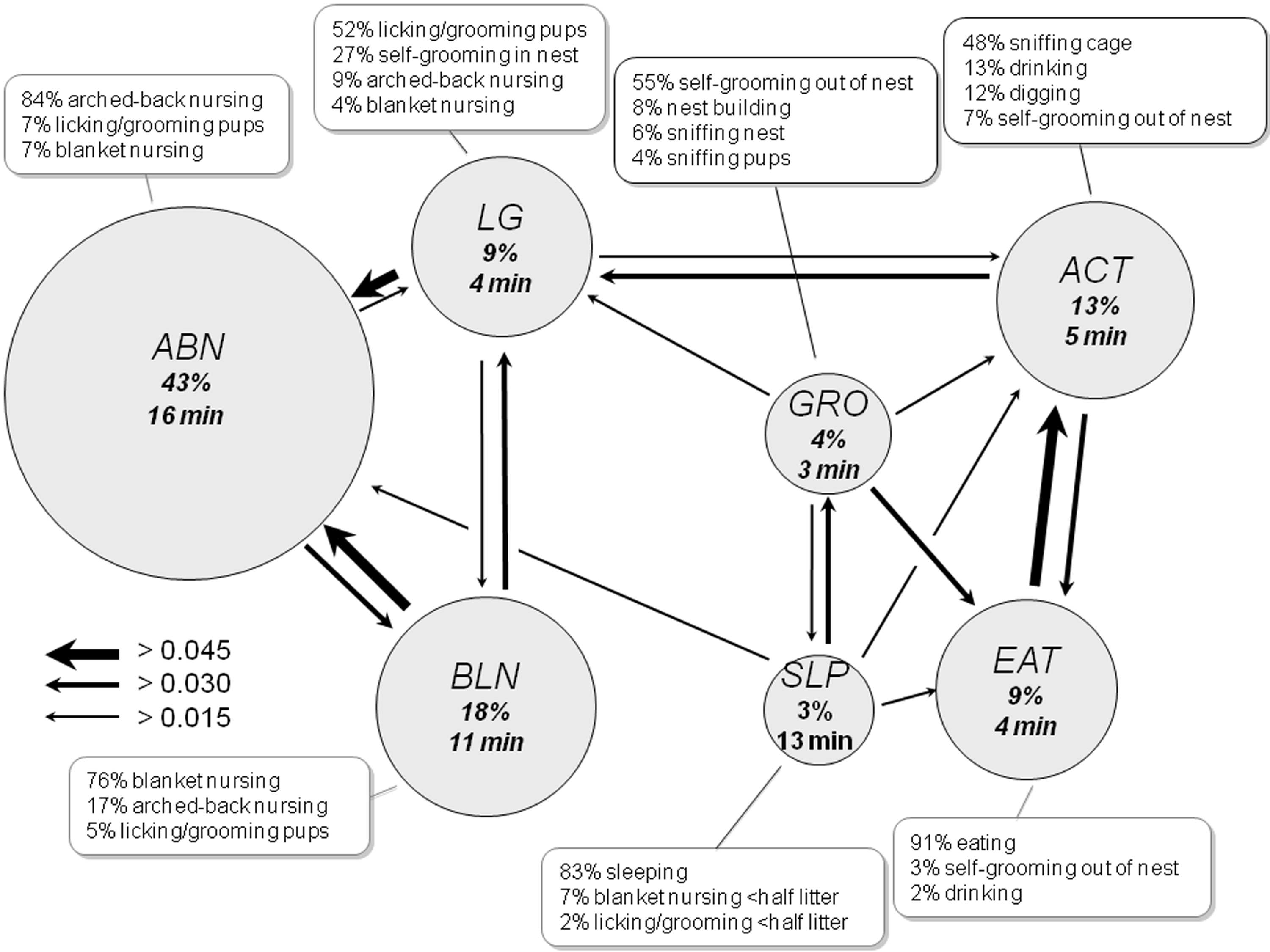

Supplement: Figure S7 — Maternal behavior strategy of CxB6 mothers. Graphical representation of composition, duration, frequency, and transition probabilities of HMM states for CxB6 mothers. Most states are composed of a single dominant behavior and multiple minor behaviors. State frequency and mean duration are indicated in site each circle. The area of each circle is proportional to state frequency. Arrows indicate absolute transition probabilities between states (transitions/minute). (1.11 MB TIF) [file pone.0014753.s020.tif]
